# A new functional JAZ degron sequence in strawberry JAZ1 revealed by structural and interaction studies on the COI1–JA-Ile/COR–JAZs complexes

**DOI:** 10.1038/s41598-020-68213-w

**Published:** 2020-07-09

**Authors:** Adrián Garrido-Bigotes, Felipe Valenzuela-Riffo, Marcela Torrejón, Roberto Solano, Luis Morales-Quintana, Carlos R. Figueroa

**Affiliations:** 1 Laboratory of Plant Epigenetics, Faculty of Forest Sciences, Universidad de Concepción, Concepción, Chile; 2 Institute of Biological Sciences, Campus Talca, Universidad de Talca, Talca, Chile; 3 Laboratory of Signaling and Development, Department of Biochemistry and Molecular Biology, Faculty of Biological Sciences, Universidad de Concepción, Concepción, Chile; 4 Plant Molecular Genetics Department, Centro Nacional de Biotecnología-CSIC (CNB-CSIC), Madrid, Spain; 5 Multidisciplinary Agroindustry Research Laboratory, Instituto de Ciencias Biomédicas, Universidad Autónoma de Chile, Talca, Chile

**Keywords:** Jasmonic acid, Plant signalling

## Abstract

The phytohormone jasmonoyl-isoleucine (JA-Ile) regulates fundamental plant processes as developmental and defense responses. JA-Ile mediates the interaction between the F-box protein COI1 (part of the SCF^COI1^ E3 ubiquitin ligase) and a JAZ repressor leading to early jasmonate responses. The Arabidopsis JAZ1 protein contains the canonical LPIARR degron sequence, which is responsible for the stabilization of the AtCOI1-JA-Ile-AtJAZ1 complex. In strawberry (*Fragaria* × *ananassa*) JAZ family was described at the transcriptional level during fruit development but the information about the interaction mode of this complex is still scarce at the molecular level. To gain insight into the strawberry JA-Ile receptor complex, we evaluated the interaction at the structural level, and protein models were built and analyzed for FaCOI1 and FaJAZ1, FaJAZ8.1, and FaJAZ10. The interaction between FaCOI1 and FaJAZ1, FaJAZ8.1 and FaJAZ10 were explored using several ligands, through molecular docking and molecular dynamics (MD) simulations, finding the strongest interaction with (+)-7-iso-JA-Ile than other ligands. Additionally, we tested interactions between FaCOI1 and FaJAZs by yeast two-hybrid assays in the presence of coronatine (COR, a JA-Ile mimic). We detected strong COR-dependent interactions between FaCOI1 and FaJAZ1. Interestingly, FaJAZ1 contains a new non-canonical (IPMQRK) functional degron sequence, in which Arg and Lys are the key residues for maintaining the interaction of the FaCOI1–COR–FaJAZ1 complex as we observed in mutated versions of the FaJAZ1 degron. Phylogenetic analysis showed that the IPMQRK degron is only present in orthologs belonging to the Rosoideae but not in other Rosaceae subfamilies. Together, this study uncovers a new degron sequence in plants, which could be required to make an alternative and functional JA-Ile perception complex in strawberry.

## Introduction

Jasmonates (JAs) are phytohormones that regulate development and stress processes in land plants^1–3^. The (+)-7-iso-jasmonoyl-l-isoleucine (JA-Ile) is the bioactive jasmonate in Arabidopsis^4^ and accumulates at the early stages of development in grape and strawberry fruits^5,6^. The bacterial phytotoxin coronatine (COR) is structurally and functionally analogous to JA-Ile^7^.

The perception mechanism of JA-Ile is mediated by the co-receptor F-box CORONATINE INSENSITIVE1 (COI1), which is part of the SKP1/CUL1/F-box (SCF^COI1^) ubiquitin E3 ligase complex^8^, and JASMONATE ZIM-DOMAIN (JAZ) repressors in Arabidopsis^9,10^. COI1, JAZ, and inositol pentakisphophate (InsP_5_) conform a three-molecule co-receptor complex essential for high-affinity JA-Ile binding^11^. Recently, using biochemical analyses Yan et al. showed that COI1 is the primary receptor recognizing JA-Ile, and subsequently interacts with JAZ proteins^12^, which are subsequently labeled with ubiquitin residues and degraded by 26S proteasome^9,10^. The formation of the COI1-JAZ co-receptor complex activates JA-signaling pathway^11^ through derepression of MYC transcription factors and the expression of downstream JA-responsive genes^9^, promoting the activation of JA-responses^9,10^ for the regulation of developmental processes or biotic and abiotic stress tolerance^3^. The JA-signaling is highly similar to the auxin-signaling pathway, specifically at receptor and repressor mechanisms^2,13^. For instance, the co-receptor COI1 is homologous to the F-box TRANSPORT INHIBITOR RESPONSE 1 (TIR1) for auxin perception^11,14,15^ and is suggested that both share a common ancestor^2,16^. COI1 amino acid sequence contains conserved leucine-rich repeats (LRRs) and F-box domains^11^. Besides, JAZ repressors are functional homologs to AUXIN/INDOLE-ACETIC ACID (Aux/IAA) repressors^13^. Arabidopsis grouping 13 members of JAZ repressors, of which JAZ1-JAZ12 contain the TIFY domain for dimerization of JAZ proteins and interaction with Novel Interactor of JAZ (NINJA) adaptor protein^17,18^. JAZ13, however, is a non-TIFY protein^19^. All Arabidopsis JAZ repressors contain a Jas domain involved in the interaction with COI1 and diverse transcription factors such as MYC, EGL, EIN, and MYB among others^3^.

The Jas domain is key for JA-Ile perception and repression of the JA-signaling pathway^1,11,17^. In Arabidopsis, most JAZ proteins contain at the N-terminal region of the Jas domain, a degron consisting in a canonical sequence LPIAR(R/K) involved in the interaction with COI1 mediated by JA-Ile^1,11,20^, however, some JAZ proteins like JAZ8 lacks this conserved sequence and they are not degraded by 26S proteasome^20,21^. The canonical degron sequence LPIAR(R/K) is conserved in most JAZ repressors in grape, tomato, apple, pear, rice, and Arabidopsis, although some proteins contain alternative variants^9,22–26^. Recently, a large amount of degron sequences has been observed in different plant lineages with great diversity in land plants^27^. Specifically, in strawberry species degron-like sequences different to canonical were reported in the woodland strawberry (*Fragaria vesca*), for instance, IPMQRK and VPQARK for FvJAZ1 and FvJAZ9, respectively. However, others like FvJAZ10 contain the canonical LPIARR sequence^20^. Besides, in silico analysis showed the functional conservation for JA-Ile perception of the IPMQRK degron of FvJAZ1^28^. Nevertheless, if this JAZ containing the IPMQRK degron sequence is functional in vivo in cultivated strawberry (*Fragaria* × *ananassa*) or if this is distinctive for *Fragaria* genus or Rosaceae family is still unknown.

Besides Arabidopsis and the bryophyte *Marchantia polymorpha*^29^, understanding of the molecular mechanisms involved in JA-Ile perception and the activation of the JA-signaling pathway in other plant species is very limited^30^. Previously, we have described the high conservation of COI1 and JAZ proteins in *F. vesca* suggesting a critical role for the JA-signaling pathway in this species^20,28^.

This study aimed was to evaluate if the new degron IPMQRK found in *F. vesca* JAZ1 is conserved in *F.* × *ananassa* and the Rosaceae family and if it can interact with the corresponding COI1 protein in the presence of COR. For this purpose, we used the strawberry JAZ and COI1 proteins, and we evaluated how this protein complex interacts with different ligands [i.e., JA-Ile, COR, and (−)-JA-Ile], combining molecular and computational strategies. The 3-D model of three strawberry JAZ (FaJAZ1, FaJAZ8.1, and FaJAZ10) proteins was constructed and used to evaluate its interaction with the FaCOI1 structural model and different ligands. After this, we evaluated FaCOI1–FaJAZs interactions in a COR-dependent manner in the yeast two-hybrid assay. The results indicate that the IPMQRK degron sequence is specific to the Rosoideae subfamily and strawberry JAZ1, which presents the non-canonical degron IPMQRK, is functional in the FaCOI1–COR–FaJAZ1 complex.

## Results

### Conservation of COI1 and JAZs in *Fragaria* × *ananassa*

To explore the conservation of *F*. × *ananassa COI1*, *JAZ1*, *JAZ8.1*, and *JAZ10,* we isolated and characterized them using the respective *F. vesca* coding sequences as templates. Protein sequences were compared with their orthologs in several model plants like apple (*Malus* × *domestica)*, grape (*Vitis vinifera)*, tomato (*Solanum lycopersicum*) and Arabidopsis (*Arabidopsis thaliana*), by multiple alignments and phylogenetic analysis*.*

Amino acid sequences were used to analyze domains and specific amino acid residues involved in the interaction of FaCOI1 with JA-Ile, InsP_5_, and JAZ repressors by multiple alignments (Supplementary Fig. S1). FaCOI1 showed the highest identity with FvCOI1 (98.6%) and MdCOI1 (81.7%), followed by VvCOI1 (77.1%) and SlCOI1 (71.6%) (Supplementary Table S1). Also, AtCOI1 and AtTIR1 exhibited the least identity values with FaCOI1, corresponding to 69.6% and 34.1%, respectively (Supplementary Table S1). FaCOI1 displayed an F-box and 18 LRR domains highly conserved concerning their ortholog proteins (Supplementary Fig. S1a). Particularly, the amino acid residues R81, R345, Y383, R406, Y441 and R493 for binding to JA-Ile, R81, R345 and R406 for binding to InsP_5_, and R345, R348, Y469 and R494 for binding to JAZ proteins, showed 100% conservation for FaCOI1 and the rest of orthologs (Supplementary Fig. S1a). Phylogenetic analysis was performed to display evolutionary relationships between COI1 proteins (Supplementary Fig. S1b). *A. thaliana* COI1 and TIR1 were clustered in group I, while *F*. × *ananassa* COI1 and the other orthologs were grouped in group II, indicating a closer phylogenetic relationship with MdCOI1, VvCOI1, and SlCOI1 (Supplementary Fig. S1b). These results suggested that FaCOI1, FvCOI1, and MdCOI1 share a common ancestor as expected since they belong to the same family (Rosaceae).

**Figure 1:**
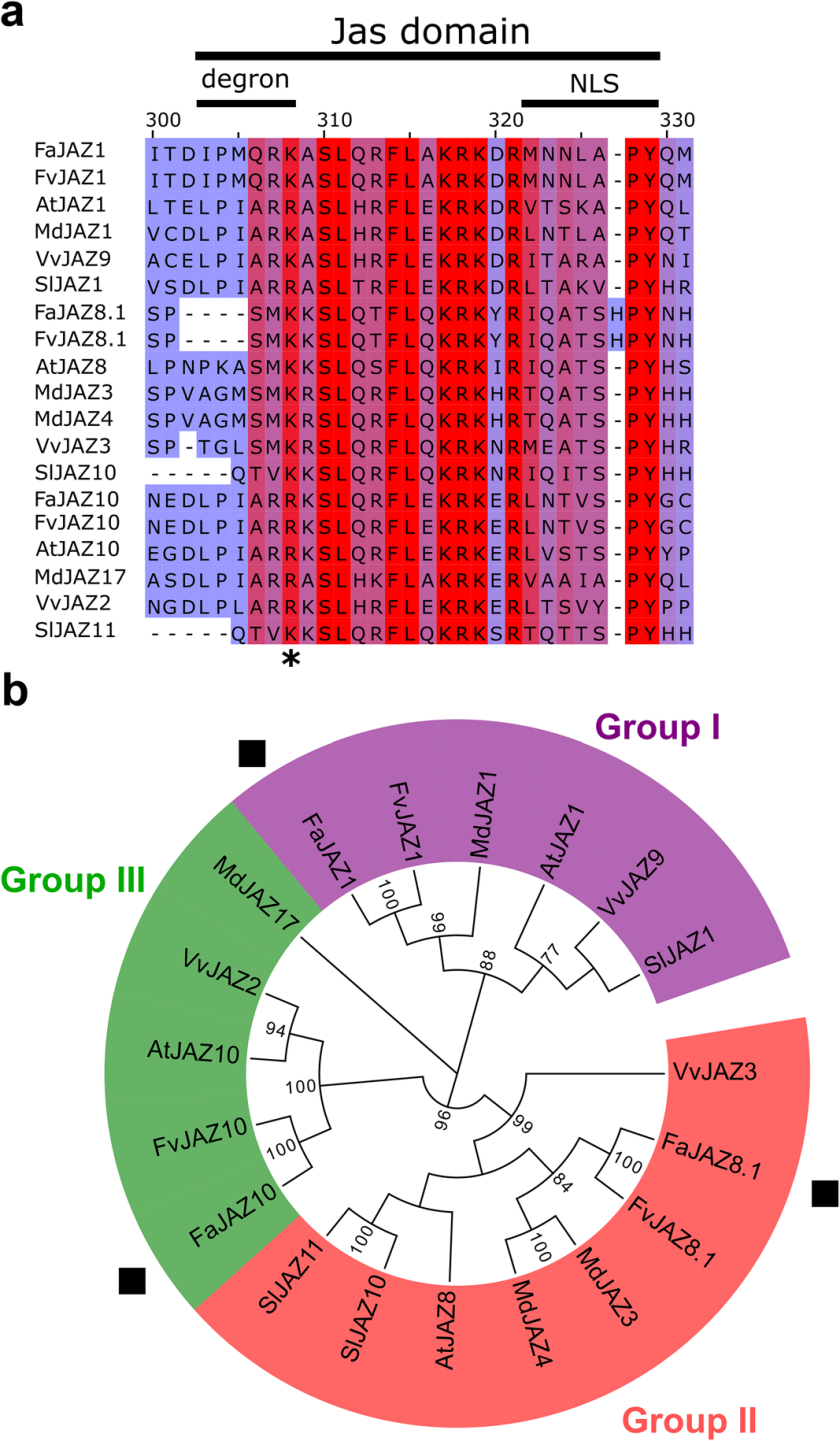
Comparison of *Fragaria* × *ananassa* JAZ1, JAZ8.1, and JAZ10 with their orthologs. Multiple alignment of Jas domain (**a**) and phylogenetic analysis for FaJAZ1, FaJAZ8.1, FaJAZ10 with their orthologs (**b**). Asterisk (*) in (**a**) denotes conserved residues for the interaction with COI1, InsP_5_, and JA-Ile according to previously reported^11^. Gaps are indicated by dashes. The red color indicates a 100% identity of amino acid residues and turns bluer with increasing amino acid diversity. Numbers in tree nodes show bootstrap values > 50%. The GenBank accession codes are available in the Material and Methods section. Fa, *Fragaria* × *ananassa*; JAZ, JASMONATE-ZIM DOMAIN.

Multiple sequence alignment of *F*. × *ananassa* JAZ1, JAZ8.1, and JAZ10 to study the conservation of domains, motifs, and amino acid residues regarding their respective orthologs was performed (Fig. 1a, b). FaJAZ1, FaJAZ8.1, and FaJAZ10 showed identities between 94–100% with their *F. vesca* orthologs (Supplementary Table S1). *M*. × *domestica* JAZ proteins exhibited the second-highest value of identity concerning FaJAZ1 (58.7%) and FaJAZ8.1 (66.4%), however, FaJAZ10 showed the least identity value respect MdJAZ17 (30.3%) (Supplementary Table S1). Finally, FaJAZ1, FaJAZ8.1, and FaJAZ10 displayed identities between 30.3 and 66.4% with apple, grape, tomato, and Arabidopsis (Supplementary Table S1). Moreover, FaJAZ1, FaJAZ8.1, and FaJAZ10 displayed high conservation of TIFY and Jas domains (Fig. 1a and Supplementary Fig. S2). For degron sequence, FaJAZ1 showed IPMQRK, like FvJAZ1; FaJAZ10 contained the canonical LPIARR, whereas FaJAZ8.1 lacked degron sequence like their orthologs (Fig. 1a, b). However, the C-terminus region of the Jas domain is highly conserved between all JAZ proteins analyzed (Fig. 1b). Phylogenetic analysis showed that FaJAZ1, FaJAZ8.1, and FaJAZ10 clustered together with their orthologs in groups I, II, and III, respectively (Fig. 1c). In summary, alignment and phylogenetic analyses indicate high conservation of *F*. × *ananassa* JAZ1, JAZ8.1, and JAZ10.

### Conservation of degron sequence IPMQRK in Rosaceae family

**Figure 2:**
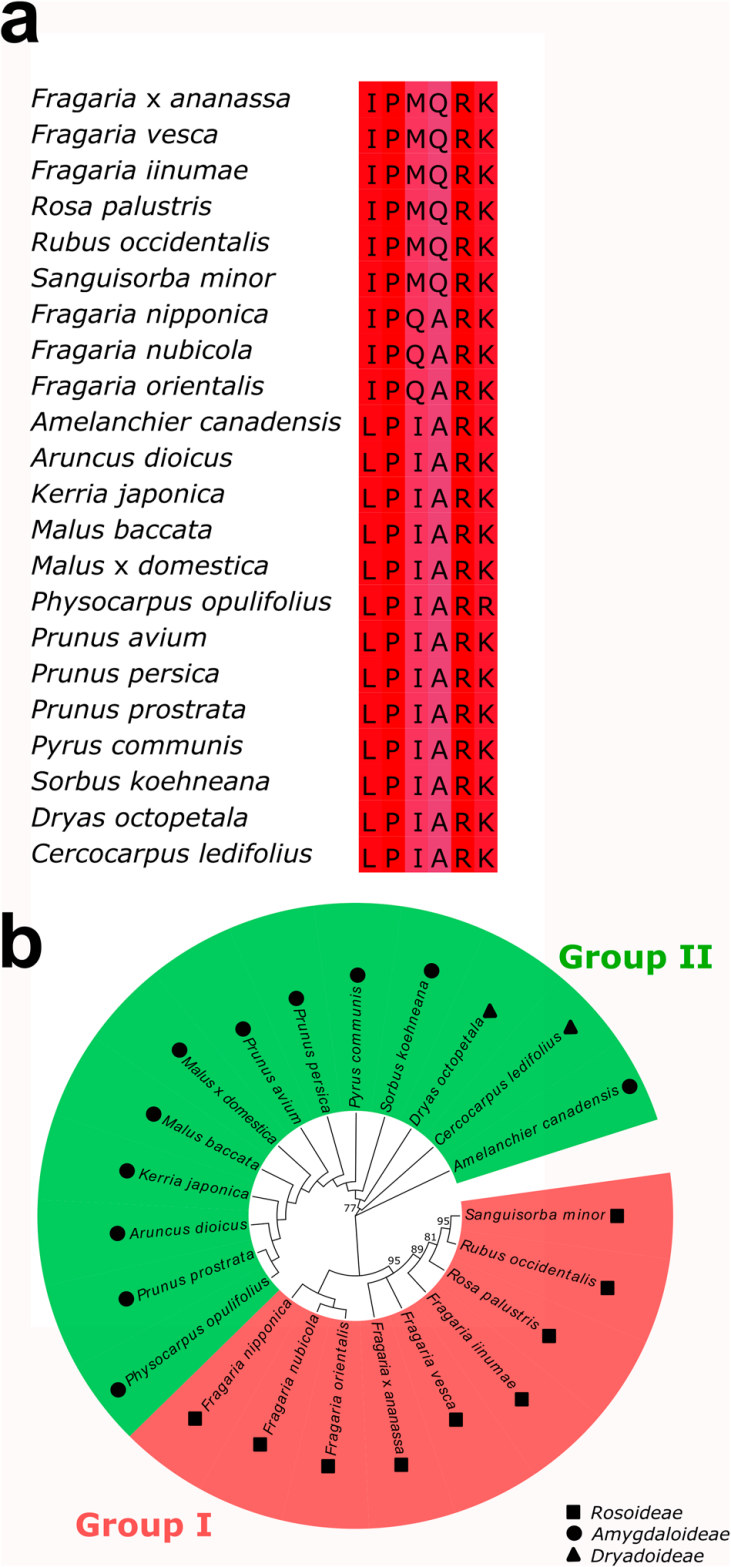
Comparison of JAZ degron sequences in the Rosaceae family. Multiple alignment of JAZ degron sequences in several species of Rosaceae family (**a**) and phylogenetic analysis of the JAZ degron sequence in the Rosaceae family (**b**). Red corresponds to 100% identity and then turns bluer with increasing amino acid residue diversity. Numbers in tree nodes show bootstrap values > 50%. Squares, circles, and triangles indicate species of Rosoideae, Amygdaloideae, and Dryadoideae subfamilies, respectively. The GenBank accession codes are available in the Material and Methods section JAZ, JASMONATE-ZIM DOMAIN.

To know if the new degron IPMQRK of FvJAZ1 and FaJAZ1 is specific of *Fragaria* genus, or if it is conserved in other plant kingdom JAZ proteins, a genome and transcriptome-wide analysis were performed (Fig. 2). First, we used IPMQRK degron sequence as a query to identify JAZ proteins with identical or highly similar degron sequence using OneKP and GDR databases. This sequence was found in *Fragaria iinumae*, *Rubus occidentalis*, *Rosa palustris,* and *Sanguisorba minor* (Fig. 2a). On the other hand, a similar sequence, IPQARK was contained in several species of *Fragaria* genus such as *F. nipponica*, *F. nubicola*, and *F. orientalis* (Fig. 2a). However, the canonical degron LPIARK was the more predominant sequence in the Rosaceae family (Fig. 2a). Degron sequences IPMQRK and IPQARK, and LPIARK were grouped in groups I and II which contains species belonging to Rosoideae, and Amygdaloideae and Dryadoideae subfamilies, respectively (Fig. 2b). These results indicate that the new degron IPMQRK is specific to the Rosoideae subfamily (Fig. 2b), suggesting an evolutionary divergence of this sequence in this subfamily.

### 3D structure of FaCOI1 and FaJAZs proteins by comparative modeling

**Figure 3:**
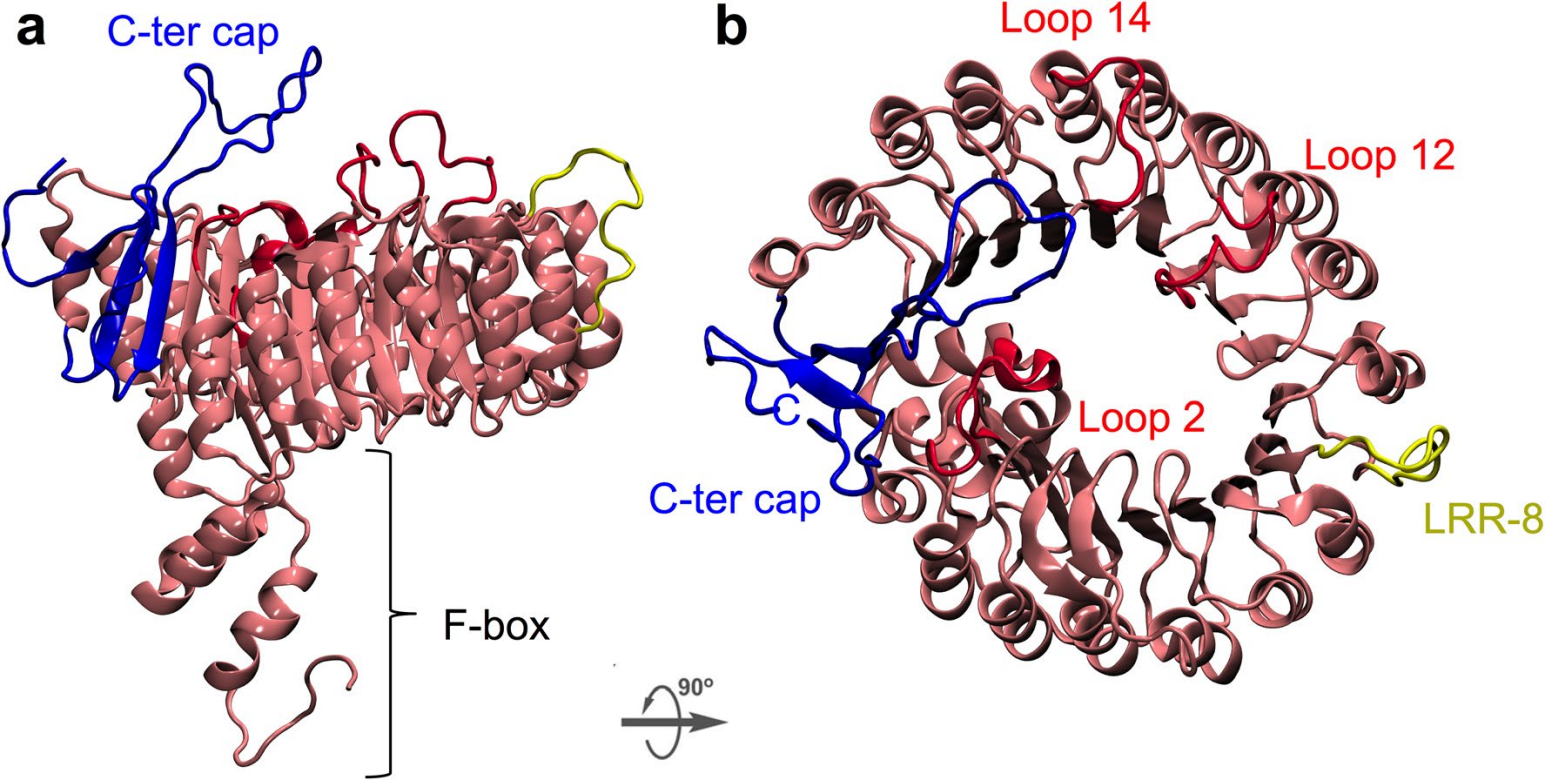
The structural model for FaCOI1. The structural model for FaCOI1 is shown as a New Cartoon representation with front (**a**) and top (**b**) views. The pink color showed the F-box and LRR domains, meanwhile in yellow color LRR-8 domain is represented. In blue color, the C-terminal is shown, and three important loops forming the JA-Ile pocket are shown in red color. Fa, *Fragaria* × *ananassa*; COI1, CORONATINE INSENSITIVE1.

To understand the molecular mechanism responsible for the interaction between FaCOI1 and three different FaJAZs proteins, we used differently in silico tools. A 3D model of FaCOI1 was built based on sequence alignment between FaCOI1 and AtCOI1 that was used as a template, the sequence alignment showed 70.5% of the sequence identity between them. To obtain a structural and energetically stable model, two optimization steps were carried out, and a short MD simulation was run to obtain a conformationally stable FaCOI1 model (Fig. 3). To validate the model structure, the structural identity was evaluated, and the RMSD values for the backbone of the FaCOI1 model, and the template was 10.11 Å (Supplementary Fig. S3). Recently, we described the structural model for FvCOI1 using a similar methodology^28^. In this sense, the RMSD of the FaCOI1 and FvCOI1 at the backbone level was 2.04 Å showing a high similarity between these two COI structures (Supplementary Fig. S3). Additionally, the stereochemical quality of the 3D model was analyzed using Ramachandran plots by the PROCHECK program. The analysis showed that the favored region was 100% (adding: most favorable regions, additionally allowed regions and generously allowed regions) indicating a good stereochemical quality (Supplementary Table S2). For an energetic approach, the FaCOI1 model showed a Z-score of − 8.83 according to ProSA2003, which was close to the value obtained for the *Arabidopsis* template (− 8.13 of Z-score). After these approaches, the final structure of FaCOI1 was structurally and energetically stable for the protein–ligand–protein analysis.

The FaCOI1 structural model was composed of two domains, which revealed a TIR1-like overall architecture^31^: the first domain corresponds to a small N-terminal tri-helical F-box domain, and the second domain is a large LRR domain (Fig. 3a). The LRR domain included seventeen LRR structures, which was oriented in a tandem structure of staggered α-helix and β-sheets (Fig. 3b). FaCOI1 showed a cavity in the center of the LRR domain, this was similar to AtTIR1 and AtCOI1^31^. This cavity was described as hormone binding or JAZ polypeptide recognized site^11^.

A hormone-dependent complex is formed by COI1 in presence of JAZs proteins with JA-Ile co-receptor^11^. To gain insights into the molecular basis of this interaction in strawberry, it was necessary the obtaining of FaJAZ tridimensional structures. For this, a search on the RCSB Protein Data Bank [based on a search of the RCSB-PDB database (https://www.rcsb.org) performed on April 28, 2020] confirmed that any X-ray crystal structure for JAZ proteins was not publicly available from Rosoideae subfamily, and structures only exist from *Arabidopsis* as AtJAZ1 Jas domain (including the degron peptide) co-crystalized with AtCOI1^11^, or the Jas-domain structure of AtJAZ9^32,33^. Also, the structural model of the FvCOI1 and FvJAZ1 from *F. vesca* has been recently reported by our group^28^. Therefore, only the Jas domain of the FaJAZ1, FaJAZ8.1, and FaJAZ10 proteins were able to model (Supplementary Fig. S4). Firstly, using AtJAZ1 as a template and our FaJAZ sequences three different sequence alignments were performed. Then, only the sequence aligned was used for each FaJAZ in each sequence alignment, so the fragment with the non-aligned sequence was removed. Two optimization steps were performed to obtain a correct model, followed by a structural and energetic evaluation (similar to the FaCOI1 model described above). The RMSD values for the backbone calculated between AtJAZ1 and the three FaJAZ structures were 4.14 Å (Supplementary Fig. S4). Additionally, the RMSD value calculated between the different FaJAZ structures was evaluated and described (Supplementary Table S3). All amino acid residues were at the favored region respect to FaJAZ1, FaJAZ8.1, and FaJAZ10 according to the PROCHECK analysis (Supplementary Table S2). Finally, the Z-score for FaJAZ1, FaJAZ8.1, FaJAZ10 was − 2.12, − 2.93, and − 1.88 respectively (Supplementary Table S2), while the AtJAZ1 (used as a template) showed a Z-score of − 1.29^28^. Thus, the final structures for FaJAZ1, FaJAZ8.1, and FaJAZ10 peptides were acceptable for protein–ligand–protein analysis.

Concerning the structural features of the three JAZ peptide fragments, the three peptides fragments adopted a similar structure, this being a bipartite structure with a loop region followed by a small α-helix for assembling with the FaCOI1–ligand complex^11,28^.

### FaCOI1–FaJAZs in silico interactions

**Figure 4:**
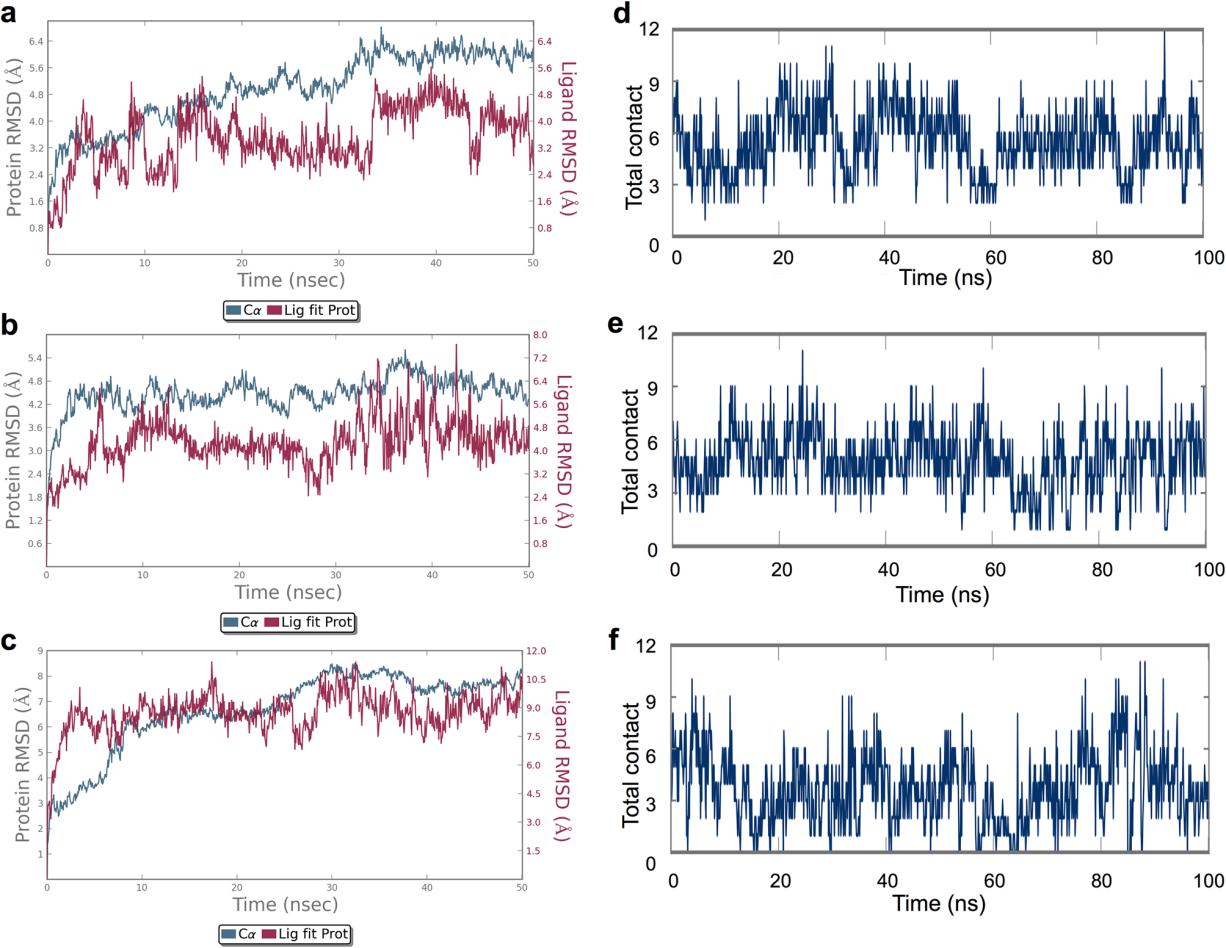
MD evaluation of the FaCOI1–FaJAZ1 complex with three different ligands. The RMSD of the Cα of the two proteins in the complex: FaCOI1–FaJAZ1 and JA-Ile (**a**), FaCOI1–FaJAZ1 and COR (**b**), and FaCOI1–FaJAZ1 and (−)-JA-Ile (**c**). Timeline representation of the total number of interactions or specific contacts (adding the H-bonds, hydrophobic interactions, ionic interactions, and water bridges) that the proteins made with the ligand throughout the MD simulation in FaCOI1–FaJAZ1 and JA-Ile (**d**), FaCOI1–FaJAZ1 and COR (**e**), FaCOI1–FaJAZ1 and (−)-JA-Ile (**f**). At, *Arabidopsis thaliana*; Fa, *Fragaria* × *ananassa*; COI1, CORONATINE INSENSITIVE1; COR, coronatine; JAZ, JASMONATE-ZIM DOMAIN.

Once the structural models were obtained, they were used to elucidate the interaction mode between FaCOI1 and the different FaJAZs structures. The initial FaCOI1–ligand–FaJAZ complexes were formed using the coordinates from the crystal structure used as a template to FaCOI1 and the different FaJAZs structures (PDB code: 3OGL) as a start point to the molecular dynamics simulations, and each complex was evaluated including the three structural components at the same time. Firstly, when we evaluated the FaCOI1–FaJAZ1 complex with JA-Ile or COR as the ligand, the two complexes were stable and showed small differences in the RMSD of the Cα, with values around 5.6 and 4.2 Å to protein complex in presence of JA-Ile and COR, respectively (blue lines in Fig. 4a, b). Now, respect to the RMSD value of each ligand, small differences were found, although in the case of the JA-Ile was slightly more stable than COR with values around 4 Å in the last 10 ns of the MD simulation, showing a low peak of 3.2 Å at the end of the MD simulation (red line in Fig. 4a). In the case of COR ligand, the RMSD value was between 4 and 4.8 Å during the last 30 ns of the simulation (red line in Fig. 4b). Respect to the (−)-JA-Ile ligand, the RMSD of the FaCOI1–FaJAZ1 complex was constant near to 8 Å, indicating that this complex is unsteady when interacting with (−)-JA-Ile (blue line in Fig. 4c). Likewise, (−)-JA-Ile ligand showed a major RMSD value respect to the other two evaluated ligands with the same protein complex, obtaining values between 8 and 10.5 Å of RMSD (red line in Fig. 4c).

As a control for the interaction between the FaCOI1–FaJAZ1 complex and the three evaluated ligands, different MD simulations were performed using the structure of the FaCOI1 and the canonical degron in AtJAZ1. The RMSD values of the Cα in the protein–protein complex were similar to those described above for FaJAZ1 in each respective ligand (blue lines in Supplementary Fig. S5) being 4 and 6 Å when these were evaluated with JA-Ile and COR, respectively. In contrast, when the ligand was (−)-JA-Ile, the value of the RMSD of the Cα was close to 9 Å. Respect to the ligands in each complex, (−)-JA-Ile was also less stable than the other two ligands (red lines in Supplementary Fig. S5). Other MD simulations with FaCOI1 and FaJAZ8.1 showed an unstable RMSD value of the Cα (greater than 12 or 13 Å) in the protein–protein complex and for the three ligands. When evaluating the AtCOI1 with FaJAZ1, the result was similar to described by FaCOI1 with AtJAZ1 with 6 Å of RMSD value, and around of 4.5 Å to with JA-Ile or COR as the ligand.

Respect to the interaction in each protein–ligand–protein complex, we observed that the total number of interactions (total contact) was similar between the FaCO1–FaJAZ1 complex with JA-Ile or COR (Fig. 4d, e). Then, when (−)-JA-Ile ligand was analyzed in the same complex the total contact was slightly smaller than the other two ligands (Fig. 4f). For FaCOI1 with AtJAZ1 the total contact was different between JA-Ile and COR, being higher in COR than JA-Ile (with values between 3 and 6 for COR and between 2 and 4 for JA-Ile) (Supplementary Fig. S5). In the case of (−)-JA-Ile, the contact was less than 2 to a great extent of the MD simulations (Supplementary Fig. S5).

**Figure 5:**
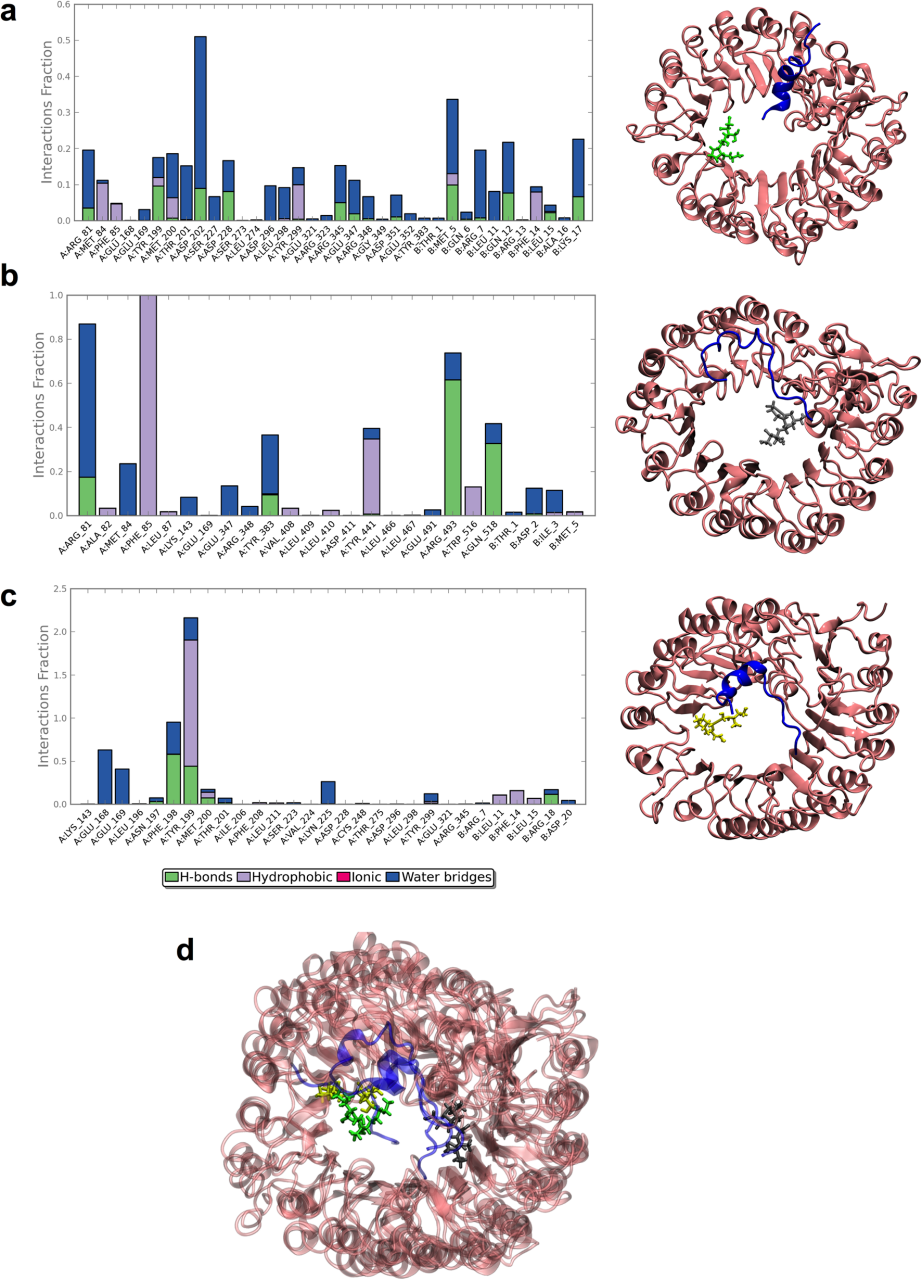
Protein–ligand interactions in each protein–ligand–protein complex. FaCOI1–FaJAZ1 and JA-Ile (**a**), FaCOI1–FaJAZ1 and COR (**b**), FaCOI1–FaJAZ1 and (−)-JA-Ile (**c**), and structural superposition of the three complexes described in a, b and c (**d**). Four types of interactions were described: the hydrogen bonds, hydrophobic interactions, ionic interactions, and water bridges are shown in green, purple, red, and blue, respectively. In the right panel, a representative view of the protein–ligand–protein interactions is shown for each complex. At, *Arabidopsis thaliana*; Fa, *Fragaria* × *ananassa*; COI1, CORONATINE INSENSITIVE1; COR, coronatine; JAZ, JASMONATE-ZIM DOMAIN.

When the residues forming the different interactions described in Fig. 4d–f were evaluated, greater differences were observed between the residues of each complex. For instance, in the case of the FaCOI1–JA-Ile–FaJAZ1 complex, FaCOI1 protein interacted with 25 residues with the other two structures, and additionally 11 residues of FaJAZ1 interacted with the JA-Ile ligand (Fig. 5a). In contrast, in the FaCOI1–COR–FaJAZ1 complex, only four residues of FaJAZ1 interact with COR ligand (Fig. 5b). In the complex FaCOI1–FaJAZ1–(−)-JA-Ile, 23 residues of FaCOI1 and six of FaJAZ1 interacted with this ligand (Fig. 5c). However, the interaction type is different in each complex, because for the JA-Ile ligand the majority of the interactions occur between the ligand and the different residues through a water molecule (i.e., forming a water bridge) (Fig. 5a). In this sense, for example, the residues Arg81, Thr201, Asp202, Asp228, Arg345, and Glu347 present the highest values of the interaction by water bridges (Fig. 5a, blue bars). In contrast, in the case of the COR ligand, the greatest values in the interactions are through hydrogen bonds (H-bonds) or hydrophobic interactions (Fig. 5b). The Phe85 residue of FaCOI1 formed a hydrophobic interaction with COR ligand throughout all MD simulations (Fig. 5b, purple bar). Other residues such as Arg81, Tyr383, and Arg493 showed high interactions mediated by water bridges, hydrophobic and H-bond interactions in a high percentage of the MD simulation time, respectively (Fig. 5b). Regarding ligand (−)-JA-Ile, the time of the MD simulation that the different residues interact with the ligand was lower than the other two simulations, and only Phe198 and Tyr199 residues showed high interaction values (Fig. 5c). Finally, no ionic interactions were observed in the three MD simulations (Fig. 5a–c). A structural superposition of the FaCOI1 with FaJAZ1 with three different ligands [COR, JA-Ile, and (−)-JA-Ile] are showed in Fig. 5d. FaJAZ1 has a similar position in the FaCOI1 cavity in the three complexes, although the ligand positions showed differences. On the one hand, JA-Ile and (−)-JA-Ile ligands oriented in a similar position inside the FaCOI1 cavity, and on the other hand COR ligand was oriented in the opposite side of the cavity respect to the other two ligands (Fig. 5d). However, the three ligands were oriented inside the FaCOI1 cavity (Fig. 5d).

Respect to the FaCOI1–AtJAZ1 complex with each ligand (Supplementary Fig. S6a–c), the results showed a similar trend to those described for complexes with FaJAZ1 described above. However, the type and interaction frequency using AtJAZ1 were lower than in the case of FaJAZ1 (Supplementary Fig. S6).

Finally, we tested other complexes using FaJAZ10 and FaJAZ8.1. For FaCOI1 in complex with FaJAZ10 and COR as a ligand (Supplementary Fig. S7a), the result was similar to described for the complex FaCOI1–AtJAZ1 (Supplementary Fig. S6), although showing lower interaction frequencies and interaction types than those observed for the FaCOI1–FaJAZ1 complex (Fig. 4b). The interaction frequencies of the FaCOI1–COR–FaJAZ8.1 complex (Supplementary Fig. S7b) were lower than the FaCOI1–COR–FaJAZ10 complex (Supplementary Fig. S7a). We used FaCOI1–COR–AtJAZ8 as a negative control (Supplementary Fig. S7c) since the AtJAZ8 Jas domain is similar to that of FaJAZ8.1.

### Protein–protein interactions of FaJAZs–FaCOI1 mediated by coronatine

**Figure 6:**
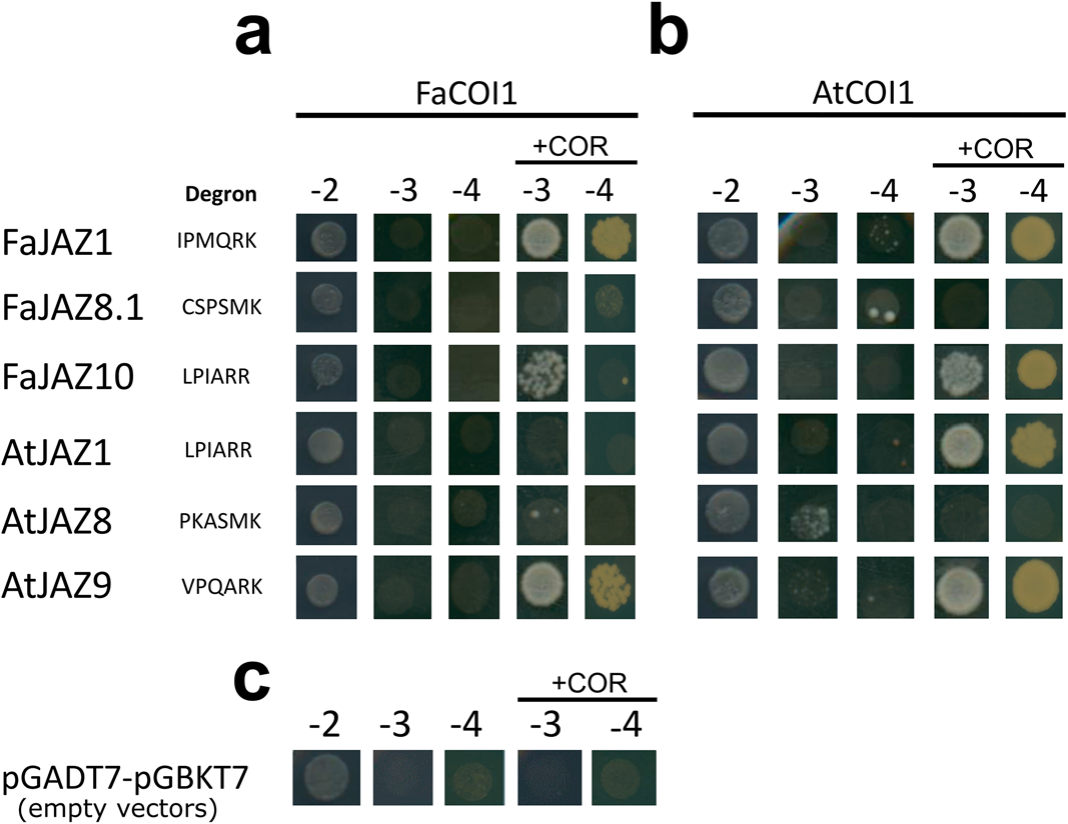
Yeast two-hybrid (Y2H) assays for JAZs-COI1s interactions using *Fragaria* × *ananassa* and *Arabidopsis thaliana* proteins. Interactions of FaCOI1 (**a**) and AtCOI1 (**b**) with JAZs repressors of *Fragaria* × *ananassa* and *Arabidopsis thaliana* under absence or presence of COR. Negative controls (**c**) with pGADT7-(AD) and pGBKT7-(DBD) empty vectors under the absence/presence of COR. − 2, SD-Leu-Trp; − 3, SD-Leu-Trp-His; − 4, SD-Leu-Trp-His-Ade; At, *Arabidopsis thaliana*; Fa, *Fragaria* × *ananassa*; COI1, CORONATINE INSENSITIVE1; COR, coronatine; JAZ, JASMONATE ZIM-DOMAIN.

**Figure 7:**
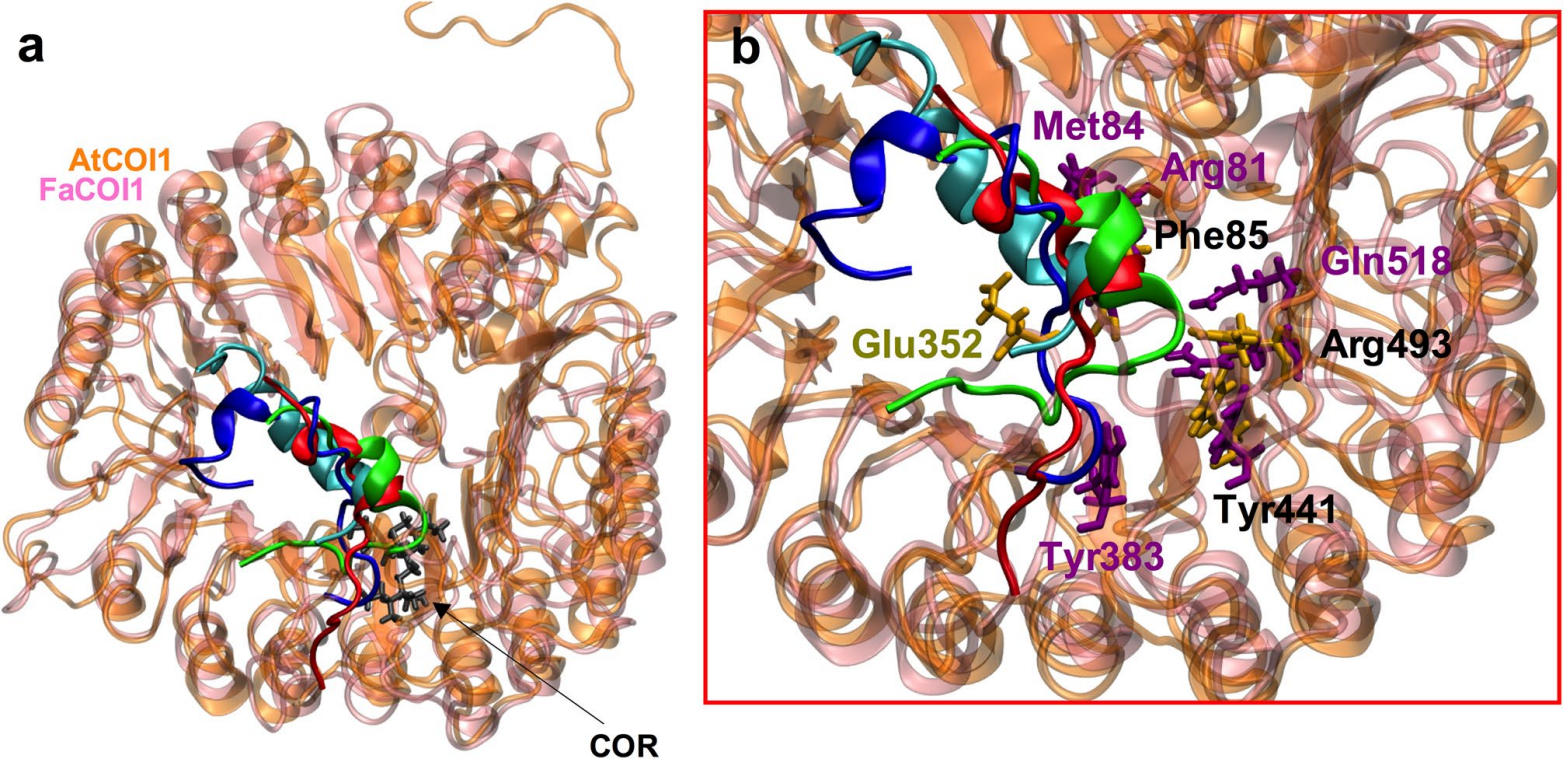
Structural superposition of the FaCOI1 and AtCOI1 after the interaction with FaJAZ1, FaJAZ10, and AtJAZ1. A representative view of the structural superposition of the four protein–ligand–protein complexes (**a**). A closer view of the FaCOI1 and AtCOI1 interaction cavity when FaJAZ1 (in blue), FaJAZ10 (in red or green), and AtJAZ1 (in cyan) are oriented in the presence of COR as a ligand in the interaction complexes (**b**). In (**b**), the main residues involved in the interaction of the AtCOI1 and FaCOI1 are showed: in yellow the residue that interacts only in the AtCOI1 with FaJAZ10 (in green) or AtJAZ1, in magenta the residues that interact only in the FaCOI1 with FaJAZ1 or FaJAZ10 (in red), and in black the residues that interact in the four complexes formed. Only for better visualization of the residues involved in the interaction, the COR ligand was removed from view in (**b**). NewCartoon representations were obtained with VMD software. At, *Arabidopsis thaliana*; Fa, *Fragaria* × *ananassa*; COI1, CORONATINE INSENSITIVE1; JAZ, JASMONATE-ZIM DOMAIN; COR, coronatine.

**Figure 8:**
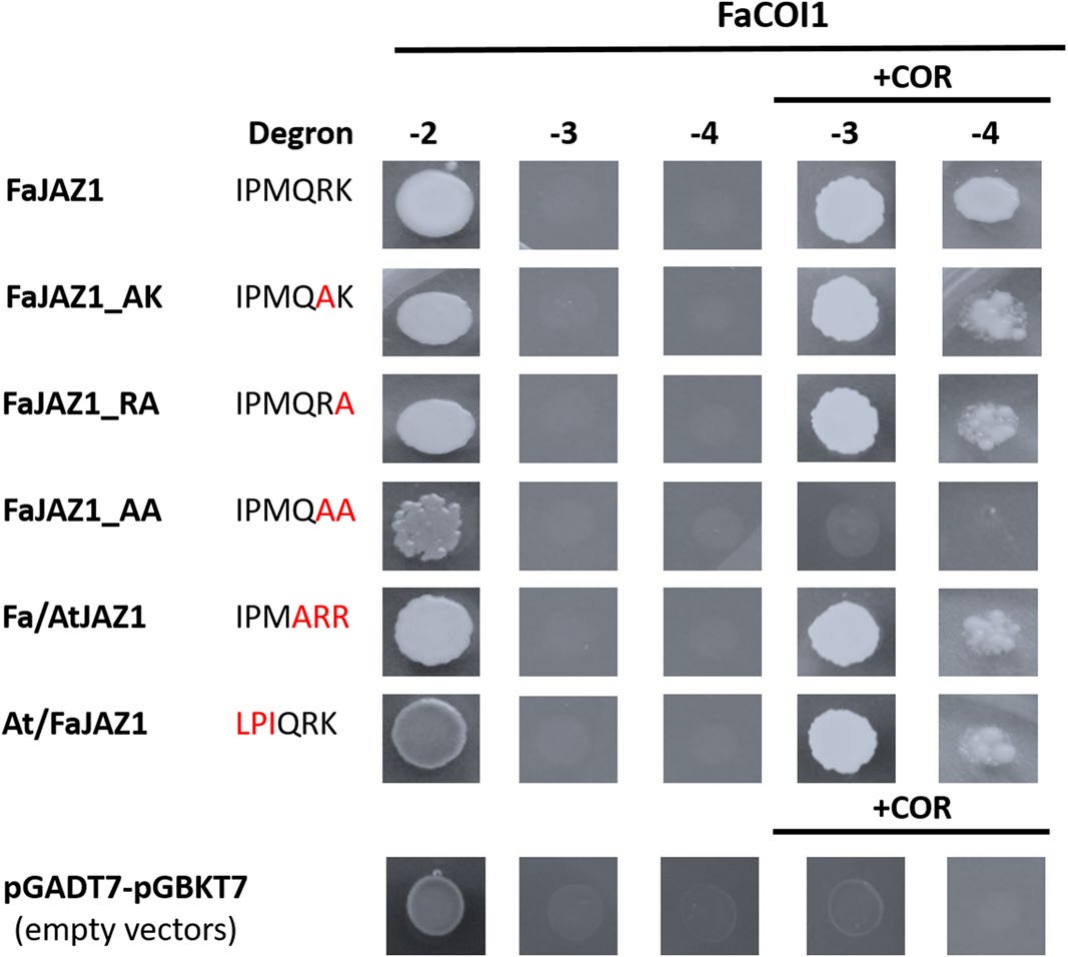
Yeast two-hybrid (Y2H) assays for *Fragaria* × *ananassa* JAZ1 mutants- and chimeras-COI1 interactions. Interactions of FaCOI1 with native (FaJAZ1), single (FaJAZ1_AK and FaJAZ1_RA) and double (FaJAZ1_AA) mutants, and chimeras (Fa/AtJAZ1 and At/FaJAZ1) of JAZ1 under absence/presence of COR. Negative controls with pGADT7-(AD) and pGBKT7-(DBD) empty vectors under the absence/presence of COR. − 2, SD-Leu-Trp; − 3, SD-Leu-Trp-His; − 4, SD-Leu-Trp-His-Ade; Fa, *Fragaria* × *ananassa*; COI1, CORONATINE INSENSITIVE1; COR, coronatine; JAZ, JASMONATE ZIM-DOMAIN.

To evaluate the formation of COI1–JAZs complexes in *F*. × *ananassa*, and to know if the degron sequence IPMQRK is functional, yeast two-hybrid (Y2H) assays on the presence/absence of COR ligand were tested (Fig. 6). Besides, the interactions AtCOI1–FaJAZs and FaCOI1–AtJAZs were performed to check the conservation of this complex formation from an evolutionary point of view (Fig. 7). Finally, we tested mutants and chimeras of the FaJAZ1 degron sequence to figure out the relevance of the degron residues in the interaction with FaCOI1 using COR as the ligand in Y2H assays (Fig. 8).

We observed that FaJAZ1 and FaJAZ10 interacted with FaCOI1 in a COR-dependent manner in SD-Leu-Trp-His selective media (Fig. 6a). However, the interaction was not observed for FaJAZ10–FaCOI1 under more restrictive conditions (SD-Leu-Trp-His-Ade) (Fig. 6a). For FaCOI1–AtJAZs interactions, only a positive interaction between AtJAZ9 and FaCOI1 promoted by COR was observed (Fig. 6a). Respect to AtCOI1–FaJAZs interactions, both FaJAZ1 and FaJAZ10 interacted with AtCOI1 in restrictive media and supplemented with COR (Fig. 6b). On the other hand, AtJAZ1 and AtJAZ9 interacted with AtCOI1 which was observed by colony growth in presence of COR (Fig. 6b). Moreover, FaJAZ8.1 and AtJAZ8 did not promote interaction with FaCOI1 or AtCOI1 under any condition (Fig. 6a, b). Finally, negative controls constituted by pGADT7-(AD) and pGBKT7-(DBD) empty vectors not exhibited activation of reporter genes (Fig. 6c). Taken together, these results suggest that the new degron sequence IPMQRK of FaJAZ1 is functional in the FaCOI1–COR–FaJAZ1 and AtCOI1–COR–FaJAZ1 complexes and the perception mechanism of the JA-signaling pathway is conserved in cultivated strawberry.

Remarkably, to evaluate the differences observed in the interaction of FaCOI1–COR–FaJAZ10 vs. AtCOI1–COR–FaJAZ10, and FaCOI1–COR–AtJAZ1 vs. AtCOI1–COR–AtJAZ1 (Fig. 6a, b), considering that those JAZ proteins present the same degron sequence (LPIARR), we performed a model with the structural superposition of FaCOI1 and AtCOI1 and the different JAZ proteins (FaJAZ1, FaJAZ10, and AtJAZ1) using COR as a ligand (Fig. 7 and Supplementary Fig. S8). Thus, we show FaJAZ10 in the interaction with AtCOI1 (in green in Fig. 7, Supplementary Fig. S8a, b), and with FaCOI1 (in red in Fig. 7, Supplementary Fig. S8c, d); AtJAZ1 in the interaction with FaCOI1 (in cyan in Fig. 7, Supplementary Fig. S8a, b), and FaJAZ1 in the interaction with FaCOI1 (in blue in Fig. 7, Supplementary Fig. S8c, d). In the structural superposition analysis, we observed a similar orientation of the different JAZs proteins when interacting with FaCOI1 or AtCOI1 (Fig. 7a). However, the interaction residues of the AtCOI1 and FaCOI1 were slightly different (Fig. 7b). Thus, the important conserved residues involved in the FaCOI1–FaJAZ1 and FaCOI1–FaJAZ10 interactions were Arg81, Met84, Phe85, Tyr383, Tyr441, Arg493 and Gln518 (Fig. 7b, Supplementary Fig. S8d), while concerning AtCOI1–FaJAZ10 and AtCOI1–AtJAZ1 were Phe85, Glu352, Tyr441 and Arg493 (Fig. 7b, Supplementary Fig. S8b). Interestingly, only three residues (Phe85, Tyr441, and Arg493) were common for the four different complexes (showed in black in Fig. 7b), meanwhile, only Glu352 was exclusively found in the formation of AtCOI1–COR–FaJAZ10 and AtCOI1–COR–AtJAZ1 complexes (Fig. 7b, Supplementary Fig. S8b), and Arg81, Met84, Tyr383, and Gln518 residues were only found in the FaCOI1–COR–FaJAZ1 and FaCOI1–COR–FaJAZ10 complexes (Fig. 7b, Supplementary Fig. S8d), suggesting that differences in residue interactions could account for the lower stability of the FaCOI1–COR–FaJAZ10 and FaCOI1–COR–AtJAZ1 complexes (Fig. 6a).

Finally, to evaluate the importance of the residues sequence of the FaJAZ1 degron in the interaction with FaCOI1 under the presence of the COR ligand, Y2H assays using JAZ1 degron mutants and chimeras were performed (Fig. 8). Single and double mutants in the degron sequences named as FaJAZ1_AK (IPMQAK), FaJAZ1_RA (IPMQRA), and FaJAZ1_AA (IPMQAA), and the chimeras At/FaJAZ1 (LPIQRK) and Fa/AtJAZ1 (IPMARR) were constructed. We observed that mutants FaJAZ1_AK, FaJAZ1_RA, and chimeras Fa/AtJAZ1 and At/FaJAZ1 present interactions in the formation of the FaCOI1–FaJAZs complex in a COR-dependent manner, although we observed weak interaction in the SD-Leu-Trp-His-Ade selection media (Fig. 8). Remarkably, the interaction of FaCOI1 with the double mutant FaJAZ1_AA does not present interaction in the formation of the complex under any selective media (Fig. 8). As a positive control, we included the native FaJAZ1, which showed strong interaction with FaCOI1 in presence of COR (Fig. 8). Besides, negative control of the empty vector constructions of pGADT7-(AD) and pGBKT7-(DBD), did not present interaction (Fig. 8). These results suggest that the Arg and Lys residues in the FaJAZ1 degron (IPMQRK) play a key role in the formation of the COI1–JAZ complex.

## Discussion

The perception mechanism of the JA-signaling pathway is well known in Arabidopsis, which is activated through the formation of the COI1–JAZ complex mediated by the JA-Ile ligand^11,12,30^. COI1 co-receptor acts like a primary receptor recognizing the ligand JA-Ile and then binding to JAZ repressors^11,12^, which are degraded by 26S proteasome^9,10^. The functionality of JA-Ile by the COI1–JAZ complex results in the activation of the signal transduction and develops the tolerance to multiple environmental constraints and the fine-tuning of development^3^. Recently, COI1 and JAZ1 co-receptors were reported in woodland strawberry^28^.

### COI1, JAZ1, JAZ8.1, and JAZ10 contain highly conserved domains in *Fragaria* × *ananassa*

LRR domains, which are involved in the interaction with JAZ repressors^11^, are conserved in woodland strawberry COI1^28^, and also highly conserved in cultivated strawberry COI1 as we showed in the present study (Supplementary Fig. S1). Besides, specific amino acid residues for binding to JA-Ile, InsP_5_, and JAZ are maintained in FaCOI1 (Supplementary Fig. S1) similar to that observed in *F. vesca*^28^ and Arabidopsis^11^. FaCOI1, FvCOI1, and MdCOI1 share a common ancestor (Supplementary Fig. S1), consistent with their species phylogenetic position, all belonging to Rosaceae family^34^. On the contrary, COI1 proteins of *V. vinifera*, *S. lycopersicum,* and *A. thaliana* are evolutionary more distant (Supplementary Fig. S1). TIR1, the auxin receptor protein, is homologous to COI1^2,14,15^, and shows the least identity to FaCOI1 (Supplementary Table S1). Thus, COI1 is conserved in *F*. × *ananassa* and evolutionarily related to their orthologs in *F. vesca* and *M*. × *domestica*.

JAZ proteins are key repressors of JA-signaling pathway^9,10^ and are part of the perception mechanism of JA-Ile in Arabidopsis^11,12^. However, JAZ repressors are also present in *F. vesca*, where 12 JAZ proteins were characterized^6^. Moreover, proteins belonging to the JAZ subfamily were also previously characterized in *M*. × *domestica*, *Pyrus pyrifolia*, *V. vinifera*, and *S. lycopersicum*^22–25^ and a large number of dicots, monocots, gymnosperms, and lower plants^27^. As in Arabidopsis, these FvJAZ proteins contain highly conserved TIFY and Jas domains^6,28^. TIFY domain includes a TIFY motif^35^, which is involved in the interaction with NINJA adaptor protein^17^ and in the dimerization of JAZ proteins^18^. This domain is conserved in FvJAZ proteins^6,28^ and FaJAZ1, FaJAZ8.1, and FaJAZ10 (Supplementary Fig. S2). The Jas domain contains a degron sequence, S-L-X2-F-X2-K-R-X2-R and nuclear location signal (NLS) for degradation, binding to transcription factors, and importing to the nucleus, respectively^11,27,36,37^. This domain is conserved in FvJAZ1, FvJAZ8.1, and FvJAZ10 repressors^6^ and their orthologs in *F*. × *ananassa* (Fig. 1a). Finally, FaJAZ1, FaJAZ8.1, and FaJAZ10 are more evolutionarily close to their *F. vesca* and *M*. × *domestica* orthologs (Fig. 1b), similar to that observed for COI1 proteins of the same Rosaceae family^28,34^.

### IPMQRK degron is a specific sequence of Rosoideae subfamily

The canonical degron sequence LPIAR(R/K) and, specifically, the last Arg residue of JAZ1 degron is crucial for binding to COI1 in Arabidopsis and subsequent degradation by 26S proteasome^11^, which corresponds to R331 residue of LPIARR sequence in FaJAZ10 (Fig. 1a) and FvJAZ10^6,28^. However, some JAZ proteins in Arabidopsis and *F. vesca* such as JAZ8 and 8.1, lack the degron sequence^6,21^ similar to that observed for the ortholog FaJAZ8.1 (Fig. 1a), which is related with higher stability and non-degradation by 26S proteasome^21^. FaJAZ1 and FvJAZ1 contain a degron sequence defined as IPMQRK (Fig. 1a)^6,28^, an alternative to the canonical sequence of AtJAZ1^11^. This degron is present in several species of Rosoideae subfamily, which along with Amydaloideae and Dryadoideae conform the Rosaceae family^34^. For instance, the IPMQRK sequence is conserved in *F. iinumae*, *F. vesca*, *R. occidentalis*, *R. palustris,* and *S. minor* (Fig. 2a). In turn, other species of *Fragaria* genus contain the degron sequence IPQARK, while the others JAZ proteins belonging to Amydaloideae and Dryadoideae subfamilies contain the major LPIAR(R/K) canonical degron sequence (Fig. 2a, b), which is also present in JAZ proteins of Rosoideae subfamily such as FvJAZ10^20^ and FaJAZ10 (Fig. 1a). In conclusion, these results suggest that IPMQRK degron emerged during the evolution in the Rosoideae subfamily.

### The structural model for FaCOI1

Recently, we proposed a structural model for FvCOI1^28^, the ortholog protein of FaCOI1, which is involved in the interaction with JAZs in *F.* × *ananassa* (Fig. 6). First, we tested the quality of the structural model using previously validated methodologies^28,38–40^. Additionally, the percentage of identity between the protein and the template was over 70% that is usually considered good for model generations^41^. Accordingly, the structure used to evaluate COI1 ability to bind three different JAZ structures was a high-quality structure (Fig. 4, Supplementary Fig. S7, and Supplementary Table S2). The result showed that FaCOI1 harbors a surface pocket in the center of the LRR domain, previously described as a potential binding site for FvJAZ1 in FvCOI1^28^ and previously in AtJAZ1 in AtCOI1^11^.

The two characteristic domains that contain FaCOI1 structure, one in the N-terminal region (named F-box domain) and one at the C-terminal region (named LRR domain) (Fig. 3 and Supplementary Fig. S1), similar to that observed in the crystal structure of AtCOI1^11^. The superposition between the template structure and FaCOI1 structural model showed a high similarity at the two domains (Supplementary Fig. S3), and a similar result was found to the superposition between FvCOI1 and FaCOI1 structural models (Supplementary Fig. S3). Interestingly, FaCOI1 did not display the LRR-8 domain integrity, because the helix conformation has been lost in this domain, as previously described in AtCOI1^11^ and FvCOI1 model structure^28^. In contrast, Yan et al. showed a computational model for the AtCOI1 structure where the LRR-8 is formed by an α-helix, proposing that the LRR domain integrity is required for the in vivo stability of AtCOI1^42^. Although our protein structural model and the template in LRR-8 do not have an α-helix structure, they are structural and energetically stable. It was observed by PROCHECK, ProSA (Supplementary Table S2), and by the analysis of the trajectory that resulting during thermodynamic equilibrations when the MD simulation of FaCOI1 was analyzed. Additionally, Valenzuela-Riffo et al. showed that the LRR-8 region is not maintained among the different analyzed sequences^28^. In the present research, we obtained in the MD simulation that LRR-8 was not required for protein–protein or protein–ligand–protein interaction in any complex analyzed, indicating that the residues present in LRR-8 are highly variable since they are not required for the interaction mechanism of FaCOI1 protein, similar to that reported for FvCOI1^28^.

### FaCOI1–ligand–FaJAZs complexes formation

The Jas domain through its degron sequence favors JA-Ile-dependent interaction between COI1 and JAZ proteins, in Arabidopsis^11^. Some structural studies displayed that the degron sequence of AtJAZ1 is part of the N-terminal region of the Jas domain and includes six highly conserved LPIARR residues that sealed the JA-Ile ligand in the COI1 binding pocket^11^. Recently, we reported that FvJAZ1 has a putative degron variant, the IPMQRK sequence^28^ displaying similar values of interaction energy when it was evaluated both in FvJAZ1 and AtJAZ1, indicating that this new degron can probably interact with FvCOI1. Here, we showed that FaJAZ1 has the same degron sequence of the FvJAZ1, and the complex constituted between FaJAZ1 and FaCOI1 was stable and interact with COR evaluated as in vivo and in silico. Additionally, we tested JA-Ile as a ligand in silico exhibiting greater protein–ligand–protein interaction during all MD simulations (Fig. 4).

Besides, we observed that H-bonds, water bridge, and hydrophobic interaction were formed between the FaJAZ1 degron and JA-Ile ligand at the residues of C terminus (QRK), whereas the N-terminal residues (IPM) interacted directly with FaCOI1 (Fig. 5a), similar to reported by Sheard et al. and Valenzuela-Riffo et al. with the canonical degron AtJAZ1 and no-canonical degron of FvJAZ1, respectively^11,28^. Other authors also found a variant in the sequence of the canonical degron. In this sense, in finger millet, the EcJAZ1 exhibited the non-canonical MPIARK sequence^43^. These authors using a similar in silico approach and five COI1 structures from five different monocot species to show that the interaction manner for these COI1 structural models generated was through the binding to JA-Ile and COR in the presence of EcJAZ1 structure. Additionally, the authors reported that the six residues of the degron sequence MPIARK were located near to the ligand molecule, suggesting a likely interaction with it^43^.

### IPMQRK is a functional degron for the COI1–JAZ1 interaction mediated by coronatine in *F*. × *ananassa*

Interestingly, a good relationship between the in vivo data in the Y2H assays and the in silico data resulting in the evaluation of the protein–ligand–protein interactions was found since the positive results of the Y2H assay agreed with high values of the different types of interactions obtained from the different MD simulations.

COI1 binding to JAZ1 depends on bioactive JA-Ile in Arabidopsis^11^, although COI1 also recognizes COR-like structures and analogous ligand molecules^4,7,11,44^, which had also been reported for in silico analysis in *F. vesca*^27^. On the one hand, FaJAZ1 and FaJAZ10, which contain degron sequence IPMQRK and LPIARR, respectively, interact with FaCOI1 in response to COR, although we observed a weak interaction with FaJAZ10 (Fig. 6a). The slight interaction observed for the FaCOI1–FaJAZ10 complex is according to in silico interactions (Supplementary Fig. S7a). Similarly, the JAZ2 repressor interacts with COI1 mediated by COR in *M*. × *domestica*^45^. In turn, AtCOI1 co-receptor interacts with FaJAZ1 and FaJAZ10, independently of the specific degron sequence (Fig. 6b), while FaCOI1 only interacts with AtJAZ9, which contain the alternative degron sequence VPQARK (Fig. 6a). Similar results were obtained for the interaction between SlCOI1–AtJAZ9 in a COR-dependent manner^44^. Unexpectedly, FaCOI1 does not interact with the AtJAZ1 which contains the canonical LPIARR sequence (Fig. 6a). Finally, FaJAZ8.1 and its ortholog AtJAZ8 do not interact with FaCOI1 or AtCOI1 under the absence/presence of COR (Fig. 6a, b), according to results previously reported in Arabidopsis, since these JAZ proteins lack of conserved degron sequence (Fig. 1b)^6,21^. On the other hand, the interactions tested with different mutated FaJAZ degron (single and double mutants based on Melotto et al.^44^, and chimeras consisted in fused degron N- and C-ter from AtJAZ1 and FaJAZ1), indicated that the two last basic residues of degron sequence are essential for the complex formation (Fig. 8) as previously reported^11^, and suggest that the amino acids of the degron N-ter (IPM) take part in the interaction strength and stability (Fig. 8). The mutants FaJAZ1_AK (IPMQAK), FaJAZ1_RA (IPMQRA), and the chimeras Fa/AtJAZ1 (IPMARR) and At/FaJAZ1 (LPIQRK) showed a weak interaction in the establishment of the FaJAZ1–FaCOI1 complex under COR presence comparing to that observed for FaCOI1–FaJAZ1 (Figs. 6a, 8), and similar to previously reported for AtJAZ1 and AtJAZ9 mutants in *A. thaliana*^44^. Regarding results of FaJAZ1 mutants and chimeras (Fig. 8) with the undetected and weak interactions observed in the FaCOI1–COR–AtJAZ1 and FaCOI1–COR–FaJAZ10 complexes, respectively (Fig. 6), it is possible that FaCOI1 needs an N-ter degron sequence other than LPIA, such as IPMQ (FaJAZ1) of VPQA (AtJAZ9) (Fig. 8), for more strong or stable interaction. Moreover, the surrounding sequences of JAZ1 degron could modify the affinity of the complex formation mediated by COR, showing different growing patterns as we observed in the Y2H assays (Figs. 6, 8). Our results suggest that FaCOI1 evolved to structural and functional specialization for the interaction with JAZ proteins (i.e. interact with FaJAZ1 but not with AtJAZ1), which contains a non-canonical degron in *F*. × *ananassa* as IPMQRK. Further studies would be important to determine the specificity grade of interactions between FaCOI1 and other FaJAZ repressors.

## Conclusions

**Figure 9:**
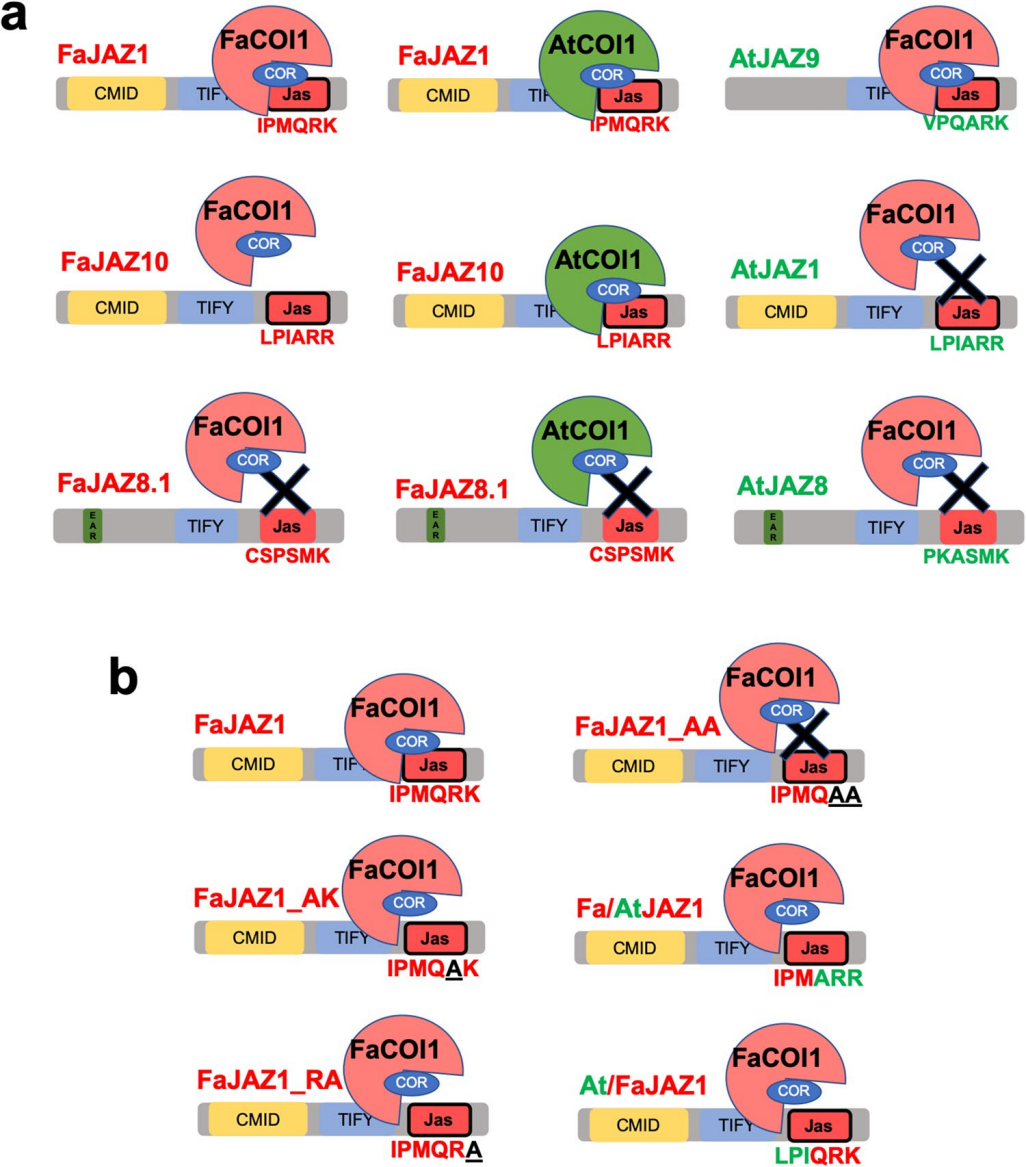
Representation of main FaCOI1–FaJAZ interactions with different JAZ degrons by the yeast two-hybrid (Y2H) system in the presence of COR performed in the present research. FaCOI1 showed strong interactions with IPMQRK (FaJAZ1) and VPQARK (AtJAZ9) degron sequences, weak and no interaction with LPIARR (FaJAZ10 and AtJAZ1, respectively) degron, and no interaction with FaJAZ8.1 and AtJAZ8 (**a**). In turn, AtCOI1 interacts strongly both with IPMQRK (FaJAZ1) and LPIARR (FaJAZ10) (**a**). When tested single and double mutants and chimeras of FaJAZ1 degron (**b**) the FaCOI1–COR–FaJAZ1 is not formed in the double mutant (FaJAZ1_AA) but a weak interaction is produced with the FaJAZ1 degron variants IPMQAK, IPMQRA, IPMARR, and LPIQRK, indicating a crucial role of Arg and Lys residues and a minor role of Ile-Pro-Met sequence of the FaJAZ1 degron in the formation of FaCOI1–COR–FaJAZ1 complex. At, *Arabidopsis thaliana*; Fa, *Fragaria* × *ananassa*; COI1, CORONATINE INSENSITIVE1; COR, coronatine; JAZ, JASMONATE ZIM-DOMAIN; CMID, Cryptic MYC2-interacting domain; EAR, ethylene-responsive element binding factor-associated amphiphilic repression; TIFY, TIFY domain.

The results of the present study are the first report on the functional characterization of the COI1–JAZ co-receptor complex in cultivated strawberry (*Fragaria* × *ananassa*) utilizing structural and experimental analyses. Previously, we described new degrons in *Fragaria vesca* JAZs^20^ and analyzed the functionality of the FvCOI1–JA-Ile–FvJAZ1 complex at computational level^28^. Now, we concluded that the new degron IPMQRK is specifically present in the Rosoideae subfamily and that *F*. × *ananassa* JAZ1, containing this degron, interacts steadily in a complex with FaCOI1 and the ligands JA-Ile or COR, as revealed by structural studies. Moreover, FaJAZ1 interacts positively with FaCOI1 and AtCOI1 in the presence of COR as revealed by protein–protein interaction studies (summarized in Fig. 9). Mutated FaJAZ1 degron at the C-ter amino acids Arg and Lys destabilized the FaCOI1–COR–FaJAZ1 complex (Fig. 9b), being both amino acids crucial for the complex formation as Arg–Arg in the canonical Arabidopsis JAZ1 degron. The N-ter IPM in the FaJAZ1 degron could be important for the complex stabilization (Fig. 9a). Remarkably, FaCOI1 could be under structural and functional specialization, since it seems to recognize better FaJAZ1 (IPMQRK) than AtJAZ1 (LPIARR) and FaJAZ10 (LPIARR). A more thorough analysis of the FaCOI1–FaJAZs interactions and the possible functional divergence of FaCOI1 could be part of further studies.

## Materials and methods

### Identification and cloning of encoding sequences for FaCOI1, FaJAZ1, FaJAZ8.1, and FaJAZ10

Full-length coding sequences of *FvCOI1* (accession code: XM_004307565), *FvJAZ1* (accession code: XM_004287607), *FvJAZ8.1* (accession code: XM_004293578) and *FvJAZ10* (accession code: XM_004310081) previously reported^6,28,46^ were used as template for primer design (Supplementary Table S4) and isolation of *F*. × *ananassa COI1* (accession code: MF511103), *FaJAZ1* (accession code: MF511104), *FaJAZ8.1* (accession code: MF511105) and *FaJAZ10* (accession code: MF511106) sequences from cDNA full-lengths of fruit. *FaCOI1* and *FaJAZ1, FaJAZ8.1,* and *FaJAZ10* full-lengths containing attBs (Supplementary Table S4) sites were recombined into pDONR207 Gateway donor vector by BP clonase II (Invitrogen). Constructs were verified by sequencing. *FaCOI1* was recombined into pGBKT7 (DNA Binding Domain, DBD) Gateway expression vector as bait and *FaJAZ1, FaJAZ8.1,* and *FaJAZ10* were recombined into pGADT7 (Activation Domain, AD) Gateway expression vector as prey, by LR clonase II (Invitrogen). These constructs were used for yeast two-hybrid (Y2H) assays.

### Sequence analysis

Predicted protein sequences of *F.* × *ananassa* (FaCOI1, FaJAZ1, FaJAZ8.1, and FaJAZ10) along with their respective orthologs in *F. vesca* (FvCOI1, FvJAZ1, FvJAZ8.1, and FvJAZ10), *Arabidopsis thaliana* (AtCOI1, AtJAZ1, AtJAZ8, and AtJAZ10), *Vitis vinifera* (VvCOI1, VvJAZ9, VvJAZ3, and VvJAZ2), *Solanum lycopersicum* (SlCOI1, SlJAZ1, SlJAZ10, and SlJAZ11) and *Malus* × *domestica* (MdCOI1, MdJAZ1, MdJAZ3, MdJAZ4, and MdJAZ17) were used for sequence analysis. A search on the RCSB Protein Data Bank (April 28, 2020) was used to confirm that X-ray crystal structure for FaCOI1, FaJAZ1, FaJAZ8.1, and FaJAZ10 proteins were not publicly available. Full-length amino acid sequences were used to perform multiple alignments using T-Coffee^47^ and visualized by Jalview software^48^. Phylogenetic analyses were conducted using the distance-based Neighbor-Joining methodology (Jones-Taylor-Thornton substitution model) and bootstrap analysis of 1,000 replicates and visualized by using ‘CLC Sequence Viewer v8.0’ (https://www.qiagenbioinformatics.com/). Finally, trees were drawn by Evolview v2 software^49^. Sequences of COI1 and JAZ1 orthologs were obtained from the previously reported^27,28^. The following GenBank accession numbers corresponding to the full-length amino acid sequences were used: FaCOI1 (*F.* × *ananassa*, ATD10398), FaJAZ1 (*F*. × *ananassa*, ATD10399), FaJAZ8.1 (*F*. × *ananassa* JAZ8.1, ATD10400), FaJAZ10 (*F*. × *ananassa* JAZ10, ATD10401), FvCOI1 (*F. vesca* COI1, XP_004307613), FvJAZ1 (*F. vesca* JAZ1, XP_004287655), FvJAZ8.1 (*F. vesca* JAZ8.1, XP_004293626), FvJAZ10 (*F. vesca* JAZ10, XP_004310129), AtCOI1 (*A. thaliana* COI1, NP_565919), AtJAZ1 (*A. thaliana* JAZ1, NP_564075), AtJAZ8 (*A. thaliana* JAZ8, NP_564349), AtJAZ10 (*A. thaliana* JAZ10, NP_001154713) VvCOI1 (*V. vinifera* COI1, AFF57759), VvJAZ9 (*V. vinifera* JAZ9, XP_002277157), VvJAZ3 (*V. vinifera* JAZ3, XP_003634826), VvJAZ2 (*V. vinifera* JAZ2, XP_002262750), SlCOI1 (*S. lycopersicum* COI1, NP_001234464), SlJAZ1 (*S. lycopersicum* JAZ1, XP_004243696), SlJAZ10 (*S. lycopersicum* JAZ8, XP_004244919), SlJAZ11 (*S. lycopersicum* JAZ11, XP_004244921.) MdCOI1 (*M*. × *domestica* COI1, XP_008392915), MdJAZ1 (*M*. × *domestica* JAZ1, XP_008388962), MdJAZ3 (*M*. × *domestica* JAZ3, XP_008371611), MdJAZ4 (*M*. × *domestica* JAZ4, XP_008371611), and MdJAZ17 (*M*. × *domestica* JAZ17, XP_008353511).

### Degron sequences analyses in Rosaceae family

Degron sequence IPMQRK was used as a query to explore its conservation in other plants of the Rosaceae family by BLASTP, using OneKP (https://www.onekp.com/) and Genome Database for Rosaceae (https://www.rosaceae.org/) databases as the subject. Protein sequence alignments were generated by T-Coffee^47^ and visualized by Jalview software^48^. Phylogenetic analyses were conducted using ‘CLC Sequence Viewer v8.0’ (https://www.qiagenbioinformatics.com/), using the Neighbor-Joining method and bootstrap analysis of 1,000 replicates, and visualized by Evolview v2 software^49^. Accession numbers in Rosaceae database: *Fragaria iinumae* (FII_iscf00051812.1.g00001.1), *Fragaria nipponica* (FNI_iscf00055213.1.g00003.1), *Fragaria nubicola* (FNU_iscf00000067.1.g00001.1), *Fragaria orientalis* (FOR_icon10232751.1.g00001.1), *Malus* × *domestica* (MD15G1220400), *Prunus avium* (Pav_sc0000129.1g1420.1.mk), *Prunus persica* (ppa009778m), *Pyrus communis* (PCP022157.1), *Rubus occidentalis* (Bras_G03023). Accession numbers in OneKP database: *Amelanchier canadiensis* (EAVM-2012760), *Aruncus dioicus* (ZPKK-2035046), *Cercocarpus ledifolius* (XFFT-2008340), *Dryas octopetala* (SQCF-2048965), *Kerria japonica* (TJQY-2055175), *Malus baccata* (VCIN-2079435), *Physocarpus opulifolius* (SXCE-2002542), *Prunus prostrata* (NCVK-2008786), *Rosa palustris* (IANR-2000625), *Sanguisorba minor* (QNOC-2003302), *Sorbus koehneana* (BLVL-2000460).

### Building the protein structures

The protein model for FaCOI1, FaJAZ1, FaJAZ8.1, and FaJAZ10 were built by a comparative modeling methodology using MODELLER 9v17 software (https://salilab.org/modeller/), according to the method described by Morales-Quintana et al.^38^. The crystal structure with PDB code of 3OGL corresponding to the COI1 protein co-crystallized with JAZ1 degron from *Arabidopsis* was selected as a template to FaCOI1, and the AtJAZ1 of this crystal was used to obtain the three different FaJAZs structural models. The SPC water model was used to build each system where the protein models were refined and structurally equilibrated, and then adding NaCl to neutralize the systems. Firstly, the four proteins (FaCOI1, FaJAZ1, FaJAZ8.1, and FaJAZ10) form an independent system, and each system was equilibrated during 10 ns by molecular dynamics simulations (MDS) and using Desmond a SCHRÖDINGER suite with OPLS v2005 force field^50^. The protein protonation state was set to pH 7.2 since this value was reported in plant cell nucleus^51^ and previously used in Valenzuela Riffo et al. to obtain the FvCOI1 and FvJAZ1 structural models^28^. To evaluate the model both ProSA2003^52^ and PROCHECK^53^ programs were employed.

### Determination of the protein–ligand interactions

First, we have positioned the FaCOI1–ligand–FaJAZ complexes using the coordinates of each structure type from the crystal structure used as a template (PDB code: 3OGL) previously to evaluate the interaction. Thus, it was possible to construct the complexes formed by FaCOI1, and FaJAZ1, FaJAZ8.1, or FaJAZ10. Each of these complexes was evaluated with three different ligands: the first one corresponds to (+)-7-iso-jasmonoyl-isoleucine (named as JA-Ile), the second one to coronatine (named as COR), and the third one to (−)-7-iso-jasmonoyl-isoleucine [named as (−)-JA-Ile], which was used as a negative control. Additionally, as positive and negative controls for the protein–protein interactions AtCOI1, AtJAZ1, AtJAZ8, and AtJAZ9 were used with the same ligands and same protocols. Then, an MD simulation for each complex was conducted. The initial coordinates of each component of the protein–ligand–protein complex used for the simulations were taken from the positioning of the three components of the protein–ligand–protein complexes described above. Each complex was built and embedded in a water box, the systems were neutralized by 0.15 M NaCl solution. Using an NVT ensemble with constant pressure (1.01325 bar) and temperature (300 K) values, each MD simulation was performed. During 100 ns of each MD simulation, only the backbone structure of FaCOI1 or AtCOI1 had a 0.25 kcal mol^−1^ Å^−2^ of the spring constant. Data were collected every 50 ps trajectory, according to the methodology previously implemented in our laboratory by Valenzuela-Riffo et al.^28^. Molecular Mechanics-Generalized Born Surface Area (MM-GBSA) analysis of the protein–ligand complex obtained in the MD simulation studies was performed. The MM-GBSA was performed using the VSGB model^54^ as an implicit solvent, and 5 Å was defined as the radius of the flexible residues. Finally, all MD simulations were analyzed using the VMD software^55^.

### Site-directed mutagenesis of the FaJAZ1 degron

Three FaJAZ1 degron mutants and two JAZ1 chimeras of *F.* × *ananassa* and *A. thaliana* were made from JAZ1 orthologs, specifically in the degron sequence IPMQRK and LPIARR using site**-**directed mutagenesis by PCR^56^. Firstly, primer sequences with muted nucleotides were designed for the construction of FaJAZ1_AK, FaJAZ1_RA, FaJAZ1_AA mutants, and At/FaJAZ1, Fa/AtJAZ1 chimeras (Supplementary Table S5). Secondly, primer sequences for the addition of attB sites used in Gateway recombination were used (Supplementary Table S4). From the coding sequence of FaJAZ1, several mutations were made specifically in the degron (IPMQRK). The first and second mutants were FaJAZ1_AK and FaJA1_RA where the amino acid Arg and Lys, respectively, were mutated to Ala (IPMQAK and IPMQRA); the third mutant was a double mutant FaJAZ1_AA, where Arg and Lys were mutated to Ala (IPMQAA). Chimeras were constructed using degron of the FaJAZ1 (IPMQRK) and AtJAZ1 (LPIARR); first chimeras was Fa/AtJAZ1, containing the first three amino acids of the FaJAZ1 (IPM) and ending with three amino acids form AtJAZ1 (ARR); the second chimera was At/FaJAZ1 using the first three amino acids from AtJAZ1 (LPI) and ending with three amino acids from FaJAZ1 (QRK).

### Yeast two-hybrid (Y2H) assays

Yeast two-hybrid (Y2H) assays were performed using the GAL4 Gateway system according to the manufacturer’s instructions (Clontech). *FaJAZ1*,* FaJAZ8.1*, *FaJAZ10*,* FaJAZ1_AK*,* FaJAZ1_RA*,* FaJAZ1_AA*,* Fa/AtJAZ1* and *At/FaJAZ1* were cloned into pGADT7 (Activation Domain, AD) Gateway expression vector and *FaCOI1* was cloned into pGBKT7 (DNA Binding Domain) Gateway expression vector. Then, these constructs were co-transformed in *Saccharomyces cerevisiae* strain AH109 to evaluate FaCOI1–FaJAZs interactions. Transformants were selected on the SD/-Leu/-Trp medium. The interactions were tested on the SD/-Leu/-Trp (− 2), SD/-Leu/-Trp/-His (− 3) and SD/-Leu/-Trp/-Ade/-His (− 4) supplemented with 50 µM COR. *AtCOI1* cloned into pGBKT7, and *AtJAZ1*, *AtJAZ8*, and *AtJAZ9* cloned into and pGADT7 were used as controls of interaction^18^. Plates were incubated at 28 °C for 5 days, respectively. The empty pGADT7 and pGBKT7 vectors were co-transformed as a negative control.

## Supplementary information

Supplementary information.
