# Supplementary material for: A new functional JAZ degron sequence in strawberry JAZ1 revealed by structural and interaction studies on the COI1–JA-Ile/COR–JAZs complexes
Source: Sci Rep. 2020 Jul 9;10:11310. doi: 10.1038/s41598-020-68213-w (PMC7347570; doi:10.1038/s41598-020-68213-w)
Supplement: Supplementary file 1 — Supplementary information. [file 41598_2020_68213_MOESM1_ESM.docx]

**Supplementary Information**

**A new functional JAZ degron sequence in strawberry JAZ1 revealed by structural and interaction studies on the COI1-JA-Ile/COR-JAZs complexes**

Adrián Garrido-Bigotes^1,+^, Felipe Valenzuela-Riffo^2,+^, Marcela Torrejón^3^, Roberto Solano^4^, Luis Morales-Quintana^5^, Carlos R. Figueroa^2,^*

^1^ Laboratory of Plant Epigenetics, Faculty of Forest Sciences, Universidad de Concepción, Concepción, Chile.

^2^ Institute of Biological Sciences, Campus Talca, Universidad de Talca, Talca, Chile.

^3^ Laboratory of Signaling and Development, Department of Biochemistry and Molecular Biology, Faculty of Biological Sciences, Universidad de Concepción, Chile.

^4^ Plant Molecular Genetics Department, Centro Nacional de Biotecnología-CSIC (CNB-CSIC), Madrid, Spain.

^5^ Multidisciplinary Agroindustry Research Laboratory, Instituto de Ciencias Biomédicas, Universidad Autónoma de Chile, Talca, Chile.

*Corresponding author:

Carlos R. Figueroa

Institute of Biological Sciences, Campus Talca, Universidad de Talca, Talca, Chile.

E-mail address: [cfigueroa@utalca.cl](mailto:cfigueroa@utalca.cl)

^+^ These authors contributed equally to this work

**Supplementary Fig. S1. Comparison of COI1 domains in *Fragaria* ×*ananassa* with their orthologs.** Multiple alignment of F-box and LRR domains (**a**) and phylogenetic analysis for FaCOI1 with their orthologs (**b**). Asterisks, open triangles, and black dots indicate conserved residues involved in JA-Ile (R81, R345, Y383, R406, Y441 and R493 in FaCOI1), InsP5 (R81, R345, and R406 in FaCOI1) and AtJAZ1 (R345, R348, Y469, and R494 in FaCOI1) interactions with AtCOI1, respectively. F-box and the 18 LRR domains are shown. Gaps are indicated by dashes. Red is 100% identity and then becomes bluer with increasing amino acid residue diversity. Nodes with bootstrap values >50% are labeled. Fa, *Fragaria* ×*ananassa*; COI1, CORONATINE INSENSITIVE1; JAZ, JASMONATE-ZIM DOMAIN.

**Supplementary Fig. S2. TIFY domain in *Fragaria* ×*ananassa* JAZ1, JAZ8.1, and JAZ10.** Gaps are indicated by dashes. Red is 100% identity and then becomes bluer with increasing amino acid residue diversity. JAZ, JASMONATE-ZIM DOMAIN.

**Supplementary Fig. S3. Structural superposition of FaCOI1 structural model and AtCOI1 or FvCOI1.** Front view of the structures of FaCOI1 and AtCOI1 (**a**), and FaCOI1 and FvCOI1 (**b**). In grey FaCOI1, in orange the template AtCOI1, and green the FvCOI1 structural. NewCartoon representations were obtained with VMD software. At, *Arabidopsis thaliana*; Fa, *Fragaria* ×*ananassa*; Fv, *Fragaria* vesca; COI1, CORONATINE INSENSITIVE1.

**Supplementary Fig. S4. FaJAZ1/8.1/10 structural models.** FaJAZ1 structural model (**a**). FaJAZ8.1 structural model (**b**). FaJAZ10 structural model (**c**). Backbone superposition between FaJAZ1 (blue color), FaJAZ8.1 (yellow color), FaJAZ10 (red color), and AtJAZ1 (cyan color) (**d**). Fa, *Fragaria* ×*ananassa*; JAZ, JASMONATE-ZIM DOMAIN.

**Supplementary Fig. S5. MD evaluation of the FaCOI1-AtJAZ1 with three different ligands.** The RMSD of the Cα of the two proteins in the complex FaCOI1-AtJAZ1 and JA-Ile (**a**), FaCOI1-AtJAZ1, and COR (**b**), FaCOI1-AtJAZ1 and (-)-JA-Ile (**c**). A timeline representation of the interactions and total contacts (H-bonds, hydrophobic interactions, ionic interactions, and water bridges) obtained during the molecular dynamics simulations and the total number of specific contacts the two proteins made with the ligand throughout the MD simulation is shown (**d-f**). FaCOI1-AtJAZ1 and JA-Ile (**d**), FaCOI1-AtJAZ1and COR (**e**), FaCOI1-AtJAZ1, and (-)-JA-Ile (**f**). At, *Arabidopsis thaliana*; Fa, *Fragaria* ×*ananassa*; COI1, CORONATINE INSENSITIVE1; COR, coronatine; JAZ, JASMONATE-ZIM DOMAIN.

**Supplementary Fig. S6. Protein-ligand interactions (or 'contacts') in each protein-protein-ligand complex**. FaCOI1-AtJAZ1 and JA-Ile (**a**), FaCOI1-AtJAZ1, and COR (**b**), FaCOI1-AtJAZ1, and (-)-JA-Ile (**c**). Four types of interaction were described; the hydrogen bonds are showed in green, the hydrophobic interactions are showed in purple, the ionic interactions are shown in red and water bridges are shown in blue. At, *Arabidopsis thaliana*; Fa, *Fragaria* ×*ananassa*; COI1, CORONATINE INSENSITIVE1; COR, coronatine; JAZ, JASMONATE-ZIM DOMAIN.

**Supplementary Fig. S7. Protein-ligand interactions (or 'contacts') in each protein-protein-ligand complex**. FaCOI1-FaJAZ10 and COR (**a**), FaCOI1-FaJAZ8.1, and COR (**b**), FaCOI1-AtJAZ8, and COR (**c**). Four types of interaction were described; the hydrogen bonds are showed in green, the hydrophobic interactions are showed in purple, the ionic interactions are shown in red and water bridges are shown in blue. At, *Arabidopsis thaliana*; Fa, *Fragaria* ×*ananassa*; COI1, CORONATINE INSENSITIVE1; COR, coronatine; JAZ, JASMONATE-ZIM DOMAIN.

**Supplementary Fig. S8. Structural superposition of the AtCOI1 with AtJAZ1 and FaJAZ10, and FaCOI1 with FaJAZ1 and FaJAZ10 using COR as the ligand.**A representative view of the structural superposition of the AtCOI1 (in orange color) interacting with AtJAZ1 (in cyan color) or FaJAZ10 (in green color) in presence of the COR (in gray color) as a ligand (**a**). A closer view of the AtCOI1 interaction cavity when FaJAZ1 or AtJAZ1 are oriented including COR as a ligand in the two complexes highlights the main residues of AtCOI1 involved in the interaction (**b**). A representative view of the structural superposition of the FaCOI1 (in pink color) interacting with FaJAZ1 (in blue color) or FaJAZ10 (in red color) in presence of the COR as a ligand (**c**). A closer view of the FaCOI1 interaction cavity when FaJAZ1 or FaJAZ10 are oriented including COR as a ligand in the two complexes highlights the main residues of FaCOI1 involved in the interaction (**d**). NewCartoon representations were obtained with VMD software. At, *Arabidopsis thaliana*; Fa, *Fragaria* ×*ananassa*; COI1, CORONATINE INSENSITIVE1; JAZ, JASMONATE-ZIM DOMAIN; COR, coronatine.

Figure S1


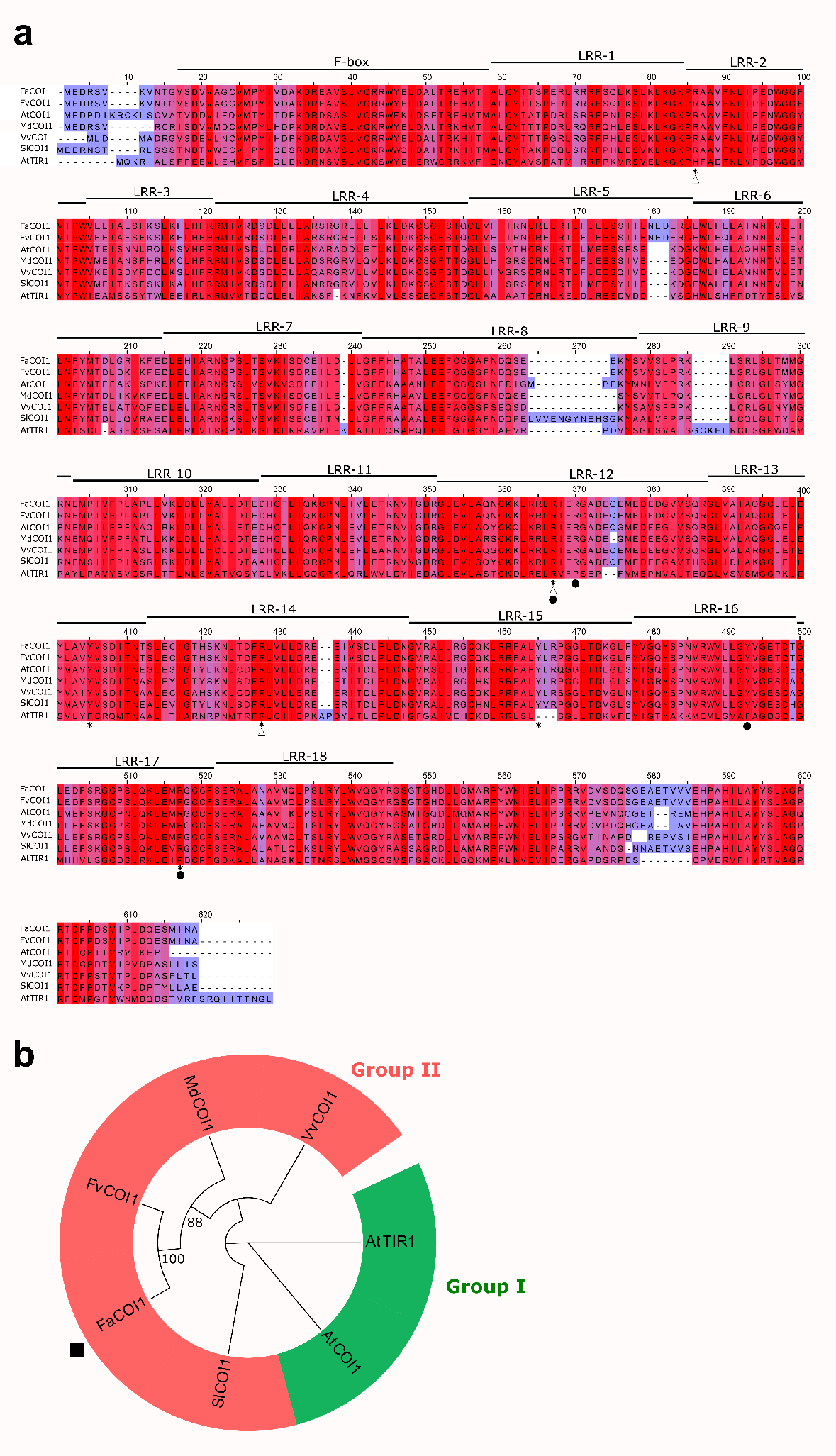


Figure S2


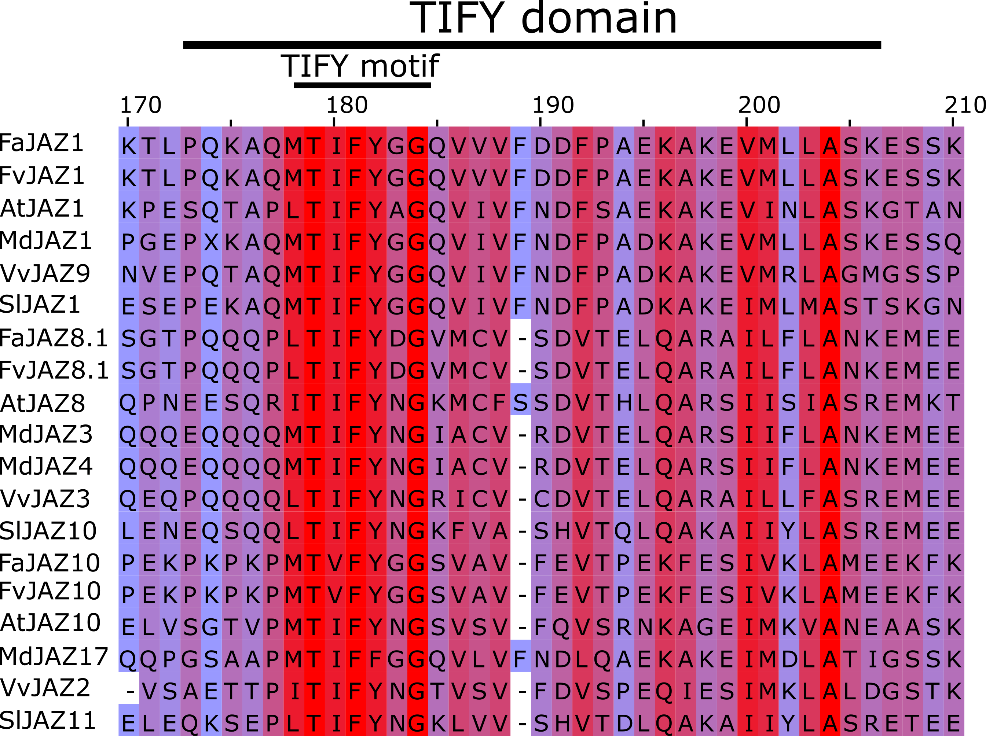


Figure S3


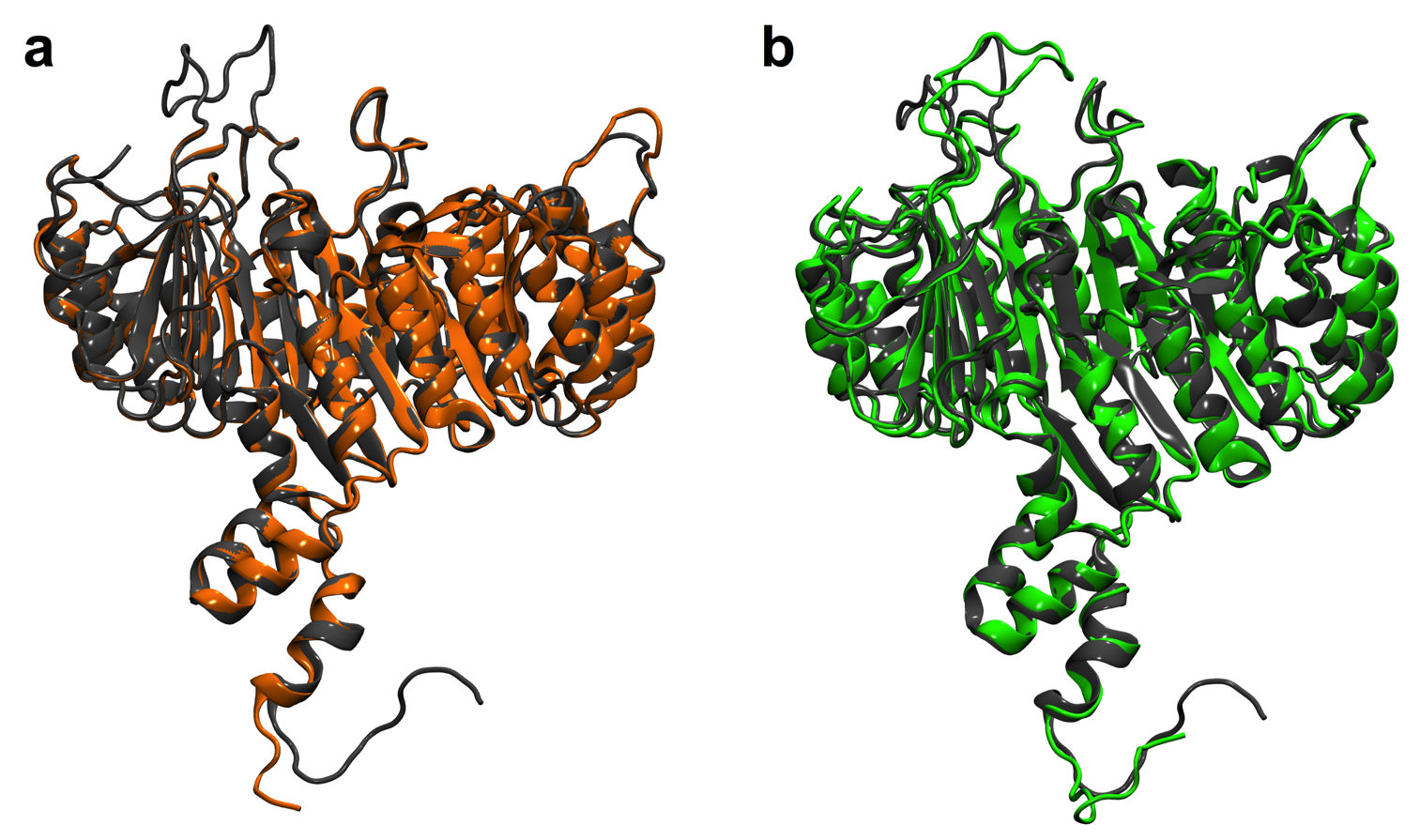


Figure S4


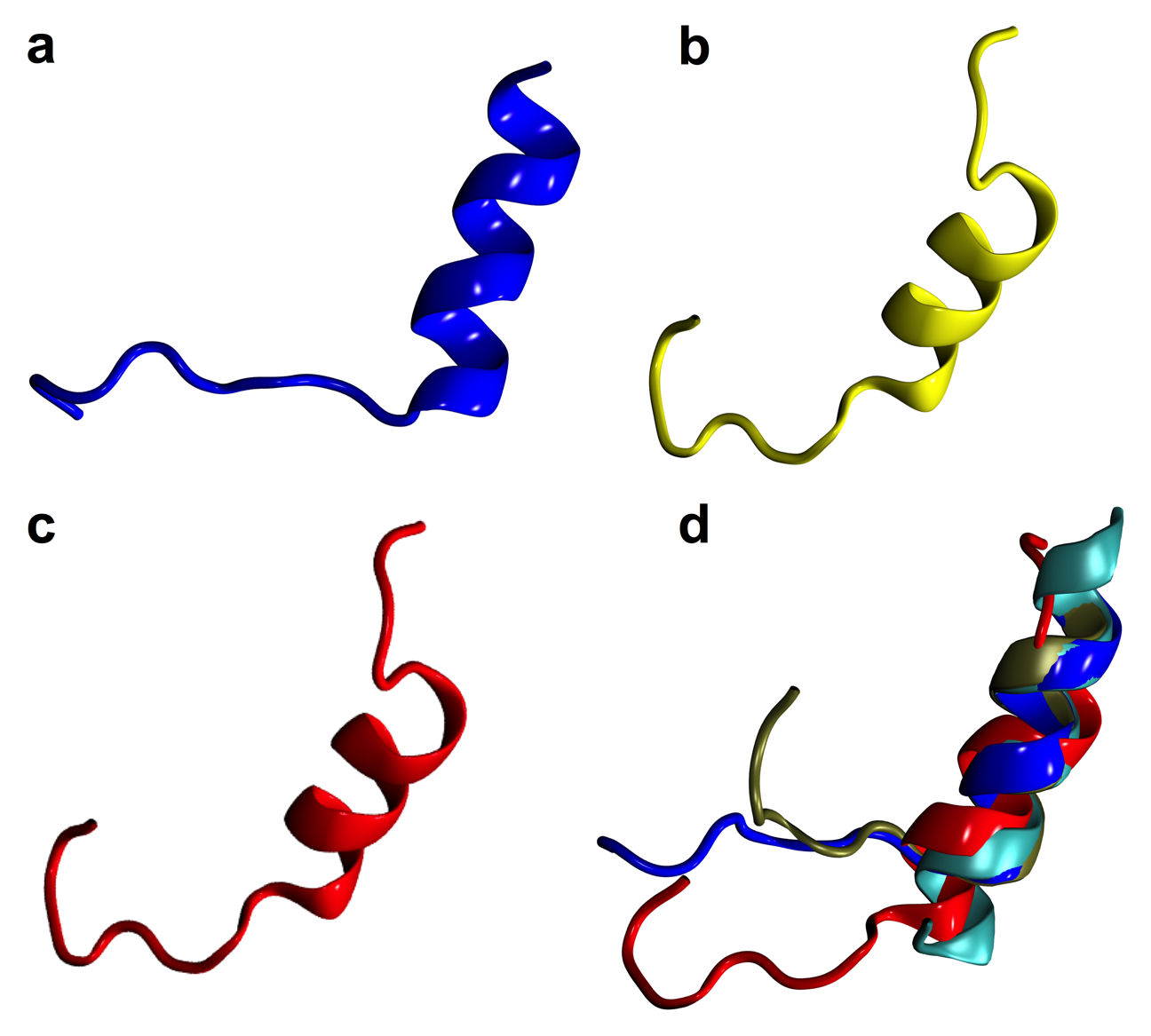


Figure S5


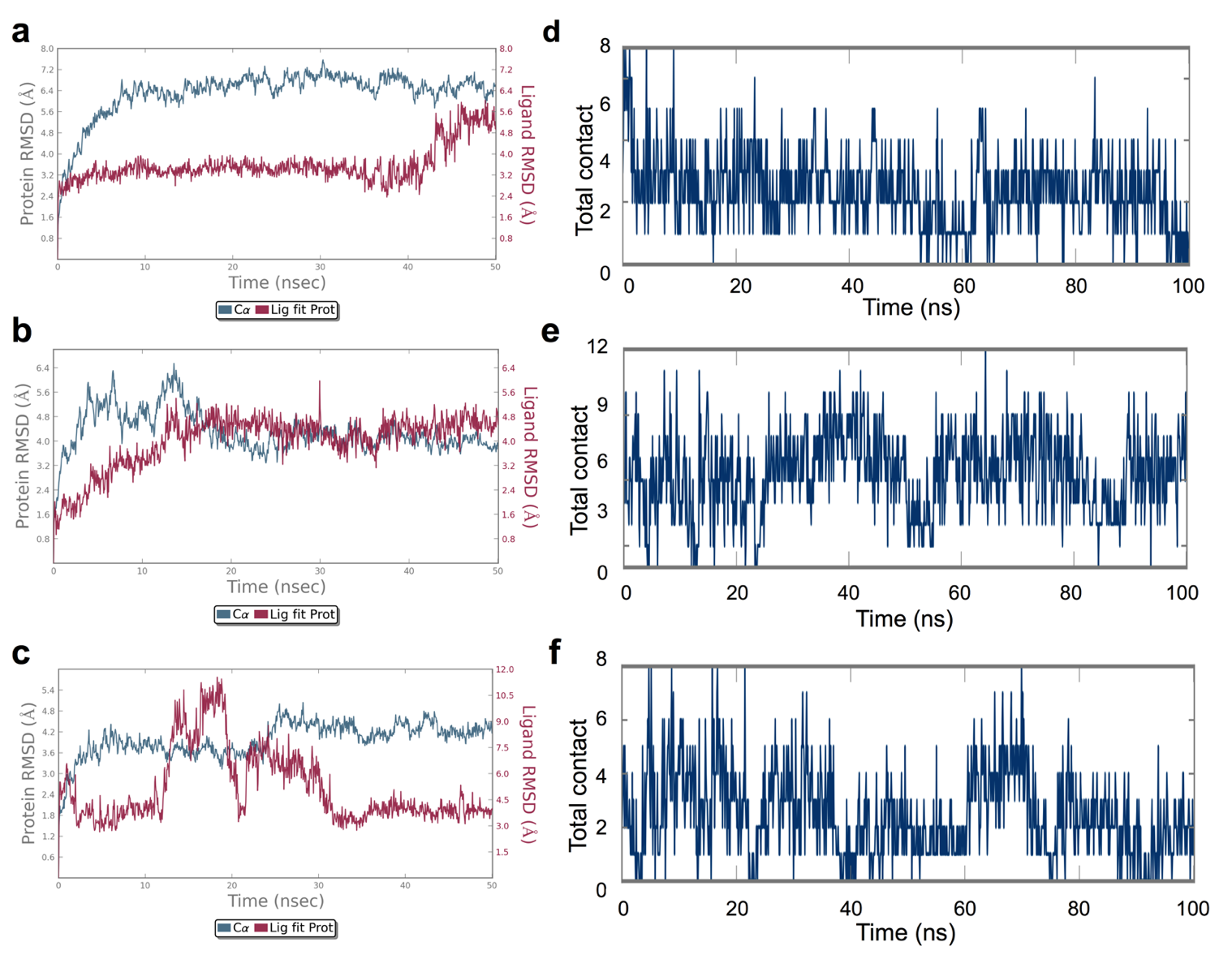


Figure S6


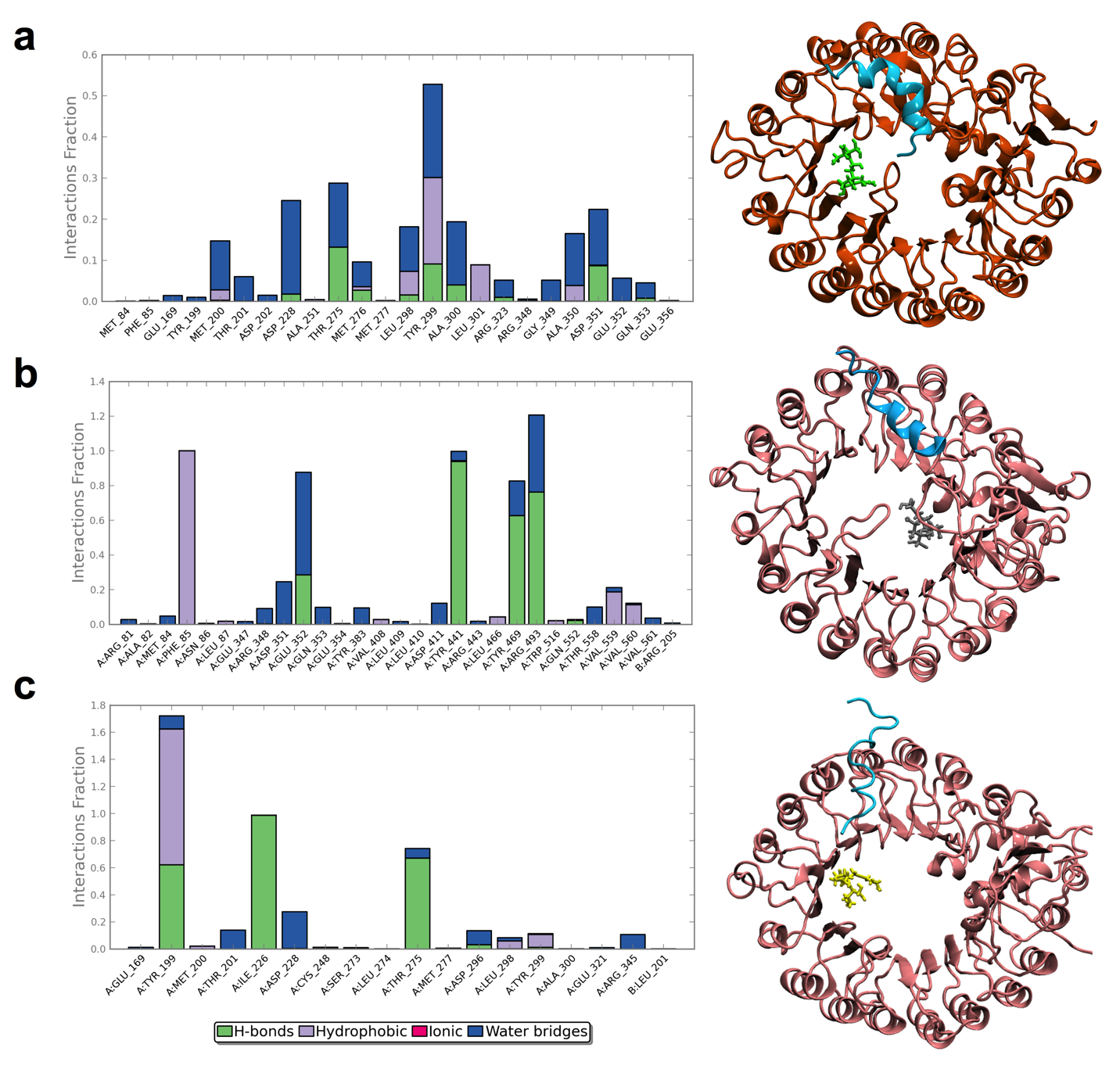


Figure S7


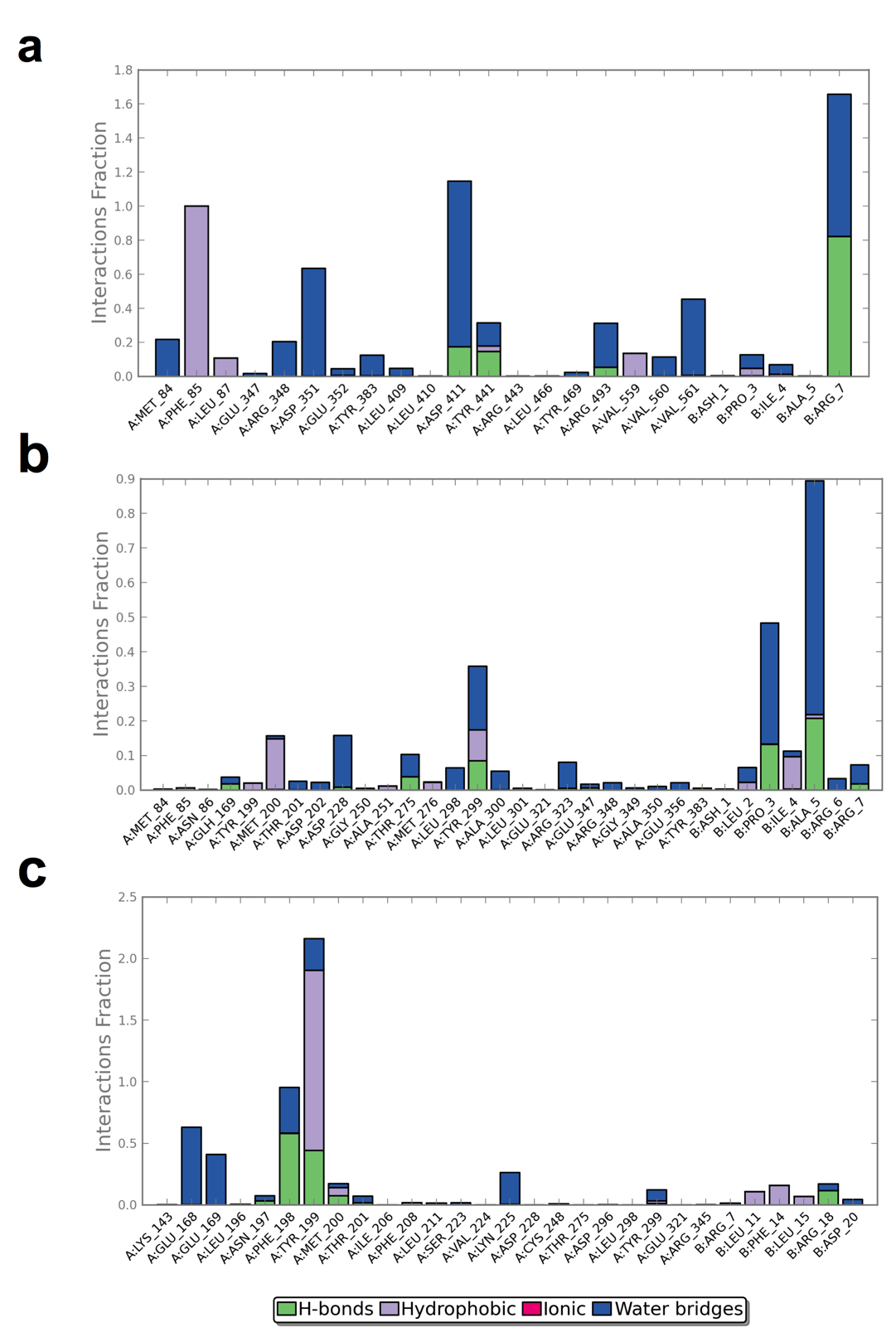


Figure S8


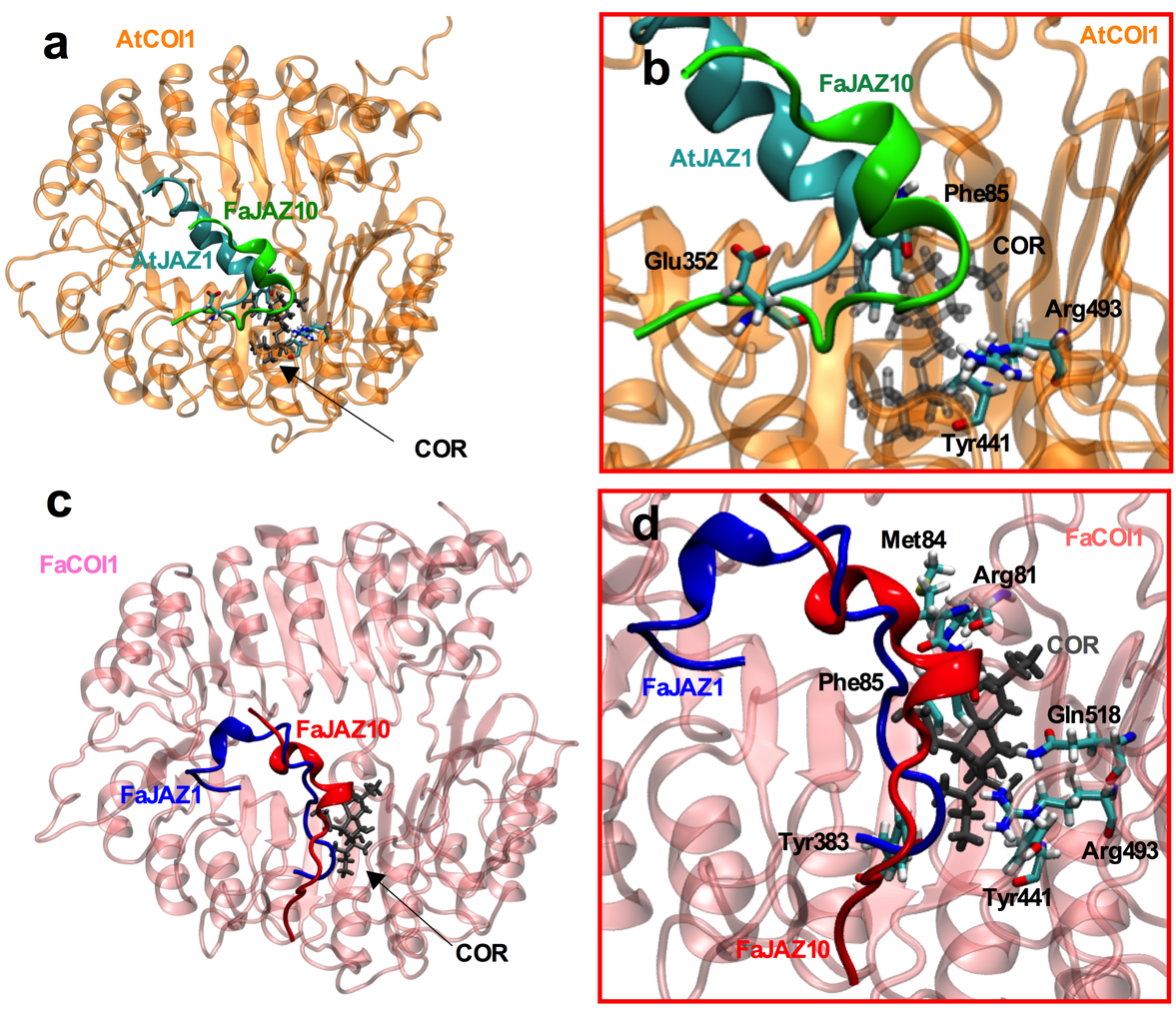


**Supplementary Table S1. Sequence identities of FaCOI1, FaJAZ1, FaJAZ8.1, and FaJAZ10 with their ortholog proteins.** Identity between *Fragaria* ×*ananassa* (Fa) COI1, JAZ1, JAZ8.1, and JAZ10 respect their *Fragaria vesca* (Fv), *Malus* ×*domestica* (Md), *Vitis vinifera* (Vv), *Solanum lycopersicum* (Sl) and *Arabidopsis thaliana* (At). AtTIR1 was included as a homologous protein for COI1. COI1, CORONATINE INSENSITIVE1; JAZ, JASMONATE-ZIM DOMAIN; TIR1, TRANSPORT INHIBITOR RESPONSE 1.

|  | **FaCOI1** | **FaJAZ1** | **FaJAZ8.1** | **FaJAZ10** |
| --- | --- | --- | --- | --- |
| FvCOI1 | 98.6 | --- | --- | --- |
| AtCOI1 | 69.6 | --- | --- | --- |
| MdCOI1 | 81.7 | --- | --- | --- |
| VvCOI1 | 77.1 | --- | --- | --- |
| SlCOI1 | 71.6 | --- | --- | --- |
| AtTIR1 | 34.1 | --- | --- | --- |
| FvJAZ1 | --- | 99.0 | --- | --- |
| AtJAZ1 | --- | 38.8 | --- | --- |
| MdJAZ1 | --- | 58.7 | --- | --- |
| VvJAZ9 | --- | 45.7 | --- | --- |
| SlJAZ1 | --- | 39.6 | --- | --- |
| FvJAZ8.1 | --- | --- | 100 | --- |
| AtJAZ8 | --- | --- | 45.9 | --- |
| MdJAZ3 | --- | --- | 66.4 | --- |
| MdJAZ4 | --- | --- | 66.4 | --- |
| VvJAZ3 | --- | --- | 61.1 | --- |
| SlJAZ10 | --- | --- | 54.5 | --- |
| FvJAZ10 | --- | --- | --- | 94.3 |
| AtJAZ10 | --- | --- | --- | 36.5 |
| MdJAZ17 | --- | --- | --- | 30.3 |
| VvJAZ2 | --- | --- | --- | 42.3 |
| SlJAZ11 | --- | --- | --- | 29.7 |

**Supplementary Table S2**. **Validation of FaCOI1, FaJAZ1, FaJAZ8.1, and FaJAZ10 protein structures using the PROCHECK program (Ramachandran plot) and ProSA analysis.** *Fragaria* ×*ananassa* (Fa). COI1, CORONATINE INSENSITIVE 1; JAZ, JASMONATE ZIM-DOMAIN.

|  | **Core (%) ^a^** | **Allow (%) ^b^** | **Gener (%) ^c^** | **Disall (%) ^d^** | **ProSA score** |
| --- | --- | --- | --- | --- | --- |
| FaCOI1 | 93.4 | 12.4 | 0.6 | 0.0 | -8.83 |
| FaJAZ1 | 100 | 0 | 0 | 0 | -2.12 |
| FaJAZ8.1 | 100 | 0 | 0 | 0 | -2.93 |
| FaJAZ10 | 100 | 0 | 0 | 0 | -1.88 |

^a^ Most favorable region; ^b^ Additionally allowed regions; ^c^ Generously allowed regions; ^d^ Disallowed regions.

**Supplementary Table S3. RMSD values (Å) of the backbone calculated between the different FaJAZ and AtJAZ1 structures.** *Arabidopsis thaliana* (At), *Fragaria* ×*ananassa* (Fa)**.** JAZ, JASMONATE ZIM-DOMAIN.

| **Structure** | **AtJAZ1** | **FaJAZ1** | **FaJAZ8.1** | **FaJAZ10** |
| --- | --- | --- | --- | --- |
| AtJAZ1 | - |  |  |  |
| FaJAZ1 | 1.8 | - |  |  |
| FaJAZ8.1 | 2.9 | 2.0 | - |  |
| FaJAZ10 | 2.1 | 1.9 | 2.1 | - |

**Supplementary Table S4. Primer sequences used for isolation of full-length coding sequences of *Fragaria* ×*ananassa* *COI1*, *JAZ1*, *JAZ8.1,* and *JAZ10*.** Red and blue colors indicate attB1 and attB2 sites, respectively, and black letters indicate specific regions for hybridization with coding sequence. *Fragaria* ×*ananassa* (Fa). attB1/attB2, sites for BP recombination; CDS, coding sequence; COI1, CORONATINE-INSENSITIVE 1; JAZ, JASMONATE ZIM-DOMAIN; bp, base pairs.

| **CDS** | **CDS (bp)** | **attB1 (5’→3’)** | **attB2 (5’→3’)** | **Amplicon size (bp)** |
| --- | --- | --- | --- | --- |
| *FaCOI1* | 1788 | GGGGACAAGTTTGTACAAAAAAGCAGGCTTGATGGAAGATCGAAGCGTC | GGGGACCACTTTGTACAAGAAAGCTGGGT**C**CTACGCATTAATCATAGACTCTTG | 1849 |
| *FaJAZ1* | 909 | GGGGACAAGTTTGTACAAAAAAGCAGGCTTGATGTCGAGCTCGTCGG | GGGGACCACTTTGTACAAGAAAGCTGGGT**C**TTACTGGGTTGGGAGAGCT | 970 |
| *FaJAZ8.1* | 393 | GGGGACAAGTTTGTACAAAAAAGCAGGCTTGATGAGGAGGAACTGCAATTT | GGGGACCACTTTGTACAAGAAAGCTGGGT**C**CTAGTGATTGTAGGGATGAGATGT | 454 |
| *FaJAZ10* | 576 | GGGGACAAGTTTGTACAAAAAAGCAGGCTTGATGTACACACCCGCCGAG | GGGGACCACTTTGTACAAGAAAGCTGGGT**C**TTAGTAGTTGTTGGTGTGACAACC | 637 |

**Supplementary Table S5. Primer sequences used for site-directed mutagenesis of *Fragaria* x *ananassa* JAZ1 degron and chimera constructions.** Black bold colors in primer sequences indicate nucleotides corresponding to the FaJAZ1 native degron. Red bold colors in primer sequences indicate nucleotides corresponding to Ala mutation. Green bold colors in primer sequences of chimeras (At/FaJAZ1 and Fa/AtJAZ1) indicate nucleotides corresponding to AtJAZ1 degron. Purple bold colors in primers sequences of chimeras (At/FaJAZ1 and Fa/AtJAZ1) indicate nucleotides corresponding to FaJAZ1 degron. Red and blue colors in Gateway adaptor primers indicate attB1 and attB2 sites, respectively, and black letters indicate specific regions for hybridization with coding sequence. JAZ, JASMONATE-ZIM DOMAIN. AtJAZ1, *Arabidopsis thaliana* JAZ1, FaJAZ, *Fragaria* x *ananassa* JAZ1.

| **FaJAZ1 mutants/chimeras** | **Fw (5’🡪3’)** | **Rv (5’🡪3’)** | **Sequence** |
| --- | --- | --- | --- |
| FaJAZ1_**A**K | GAT**ATACCAATGCAAGCGAAA**GCTTCACTGCAGCG | CGCTGCAGTGAAGCTTTCGCTTGCATTGGTATATC | **IPMQAK** |
| FaJAZ1_R**A** | GAT**ATACCAATGCAAAGGGCA**GCTTCACTGCAGCG | CGCTGCAGTGAAGCTGCCCTTTGCATTGGTATATC | **IPMQRA** |
| FaJAZ1_**AA** | GAT**ATACCAATGCAAGCGGCA**GCTTCACTGCAGCG | CGCTGCAGTGAAGCTGCCGCTTGCATTGGTATATC | **IPMQAA** |
| **At**/**Fa**JAZ1 | GAT**CTTCCTATTCAAAGGAAA**GCTTCACTGCAGCG | CGCTGCAGTGAAGCTTTCCTTTGAATAGGAAGATC | **LPIQRK** |
| **Fa**/**At**JAZ1 | GAT**ATACCAATGGCTAGAAGA**GCTTCACTGCAGCG | CGCTGCAGTGAAGCTCTTCTAGCCATTGGTATATC | **IPMARR** |
